# Supplementary material for: Brainstem fMRI signaling of surprise across different types of deviant stimuli
Source: Cell Rep. 2023 Nov 10;42(11):113405. doi: 10.1016/j.celrep.2023.113405 (PMC10698303; doi:10.1016/j.celrep.2023.113405)
Supplement: Document S1. Figures S1–S8 and Tables S1–S3 [file mmc1.pdf]

Cell Reports, Volume *42*

Supplemental information

Brainstem fMRI signaling of surprise

across different types of deviant stimuli

Audrey Mazancieux, Franck Mauconduit, Alexis Amadon, Jan Willem de Gee, Tobias H. Donner, and Florent Meyniel

Supplemental information

Table S1. Maximum Cohen's d for significant clusters after FWE correction (pFWE < 0.05) and coverage for each parcel (i.e. region) of the Destrieux parcellation. Bold font indicates the regions included in the functional connectivity analysis (i.e., more than 50% coverage). Blue regions are regions included in the manuscript.

Code	Region	Coverage	Maximum Cohen'd
**1**	**Frontomarginal gyrus and sulcus**	**0.99**	**0**
**2**	**Inferior occipital gyrus and sulcus**	**1**	**0**
3	Paracentral lobule and sulcus	0	0
**4**	**Subcentral gyrus and sulcus**	**0.52**	**0.96**
**5**	**Transverse frontopolar gyrus and sulcus**	**0.58**	**0**
6	Anterior cingulate gyrus and sulcus	0.05	0
7	Middle-anterior cingulate gyrus and sulcus	0	0
8	Middle-posterior cingulate gyrus and sulcus	0.01	0
**9**	**Posterior-dorsal part of the cingulate gyrus**	**0.55**	**0**
**10**	**Posterior-ventral part of the cingulate gyrus**	**1**	**0**
**11**	**Cuneus**	**0.65**	**1.21**
12	Opercular part of the inferior frontal gyrus	0.34	1.70
13	Orbital part of the inferior frontal gyrus	0.42	**0.52**
14	Triangular part of the inferior frontal gyrus	0.03	0.42
15	Middle frontal gyrus	0.06	0.57
16	Superior frontal gyrus	0.03	0
**17**	**Long insular gyrus and central sulcus of the insula**	**0.98**	**0.65**
**18**	**Short insular gyrus**	**0.72**	**1.93**
**19**	**Middle occipital gyrus**	**0.75**	**0**
20	Superior occipital gyrus	0.08	0
21	Lateral occipito-temporal gyrus	1	0
**22**	**Lingual gyrus**	**1**	**0.97**
**23**	**Parahippocampal gyrus**	**1**	**0**
**24**	**Orbital gyrus**	**0.67**	**0.46**
**25**	**Angular gyrus**	**0.79**	**0**
**26**	**Supramarginal gyrus**	**0.55**	**1.14**
27	Superior parietal lobule	0.06	1.24
28	Postcentral gyrus	0.001	0
29	Precentral gyrus	0.001	1.17
**30**	**Precuneus**	**0.51**	**0**
**31**	**Rectus gyrus**	**0.72**	**0**
**32**	**Subcallosal gyrus**	**0.98**	**0.70**
**33**	**Anterior transverse temporal gyrus**	**1**	**1.30**
**34**	**Lateral aspect of the superior temporal gyrus**	**1**	**2.04**
**35**	**Planum polare of the superior temporal gyrus**	**1**	**1.21**
**36**	**Superior temporal gyrus**	**0.96**	**1.12**
**37**	**Inferior temporal gyrus**	**1**	**0.97**
**38**	**Middle temporal gyrus**	**1**	**0.45**
39	Horizontal ramus of the anterior segment of the lateral sulcus	0.01	0.90
**40**	**Vertical ramus of the anterior segment of the lateral sulcus**	**1**	**0**
**41**	**Posterior ramus of the lateral sulcus**	**0.95**	**0.84**
42	Occipital pole	0.46	0.96
**43**	**Temporal pole**	**1**	**0**
**44**	**Calcarine sulcus**	**1**	**1.29**

45	Central sulcus	0.006	0.79
46	Marginal branch (or part) of the cingulate sulcus	0.04	0
**47**	**Anterior circular insula sulcus**	**0.65**	**1.79**
**48**	**Inferior circular insula**	**1**	**1.31**
49	Superior segment of the circular sulcus of the insula	0.19	1.36,
**50**	**Anterior transverse collateral sulcus**	**1**	**0**
**51**	**Posterior transverse collateral sulcus**	**1**	**0**
52	Interior frontal sulcus	0.15	0.68
53	Middle frontal sulcus	0.08	0.47
54	Superior frontal sulcus	0	0
**55**	**Sulcus intermedius primus**	**1**	**1.02**
**56**	**Intraparietal sulcus and transverse parietal sulci**	**0.70**	**1.69**
**57**	**Middle occipital sulcus and lunatus sulcus**	**0.91**	**0.49**
**58**	**Superior occipital sulcus and transverse occipital sulcus**	**0.86**	**0.47**
**59**	**Anterior occipital sulcus**	**1**	**0**
**60**	**Lateral occipito-temporal sulcus**	**1**	**0**
**61**	**Medial occipito-temporal sulcus and lingual sulcus**	**1**	**0**
62	Lateral orbital sulcus	0.01	0.61
**63**	**Medial orbital sulcus**	**0.99**	**1.28**
**64**	**Orbital sulci (H-shaped sulci)**	**0.53**	**0.67**
**65**	**Parieto-occipital sulcus**	**0.96**	**0.64**
**66**	**Pericallosal sulcus**	**0.52**	**1.08**
67	Postcentral sulcus	0.05	1.61
68	Interior precentral sulcus	0.005	2.26
69	Superior precentral sulcus	0	0
70	Suborbital sulcus	0.38	0.53
**71**	**Subparietal sulcus**	**0.72**	**0**
**72**	**Interior temporal sulcus**	**1**	**0.91**
**73**	**Superior temporal sulcus**	**1**	**1.48**
**74**	**Transverse temporal sulcus**	**1**	**1.57**

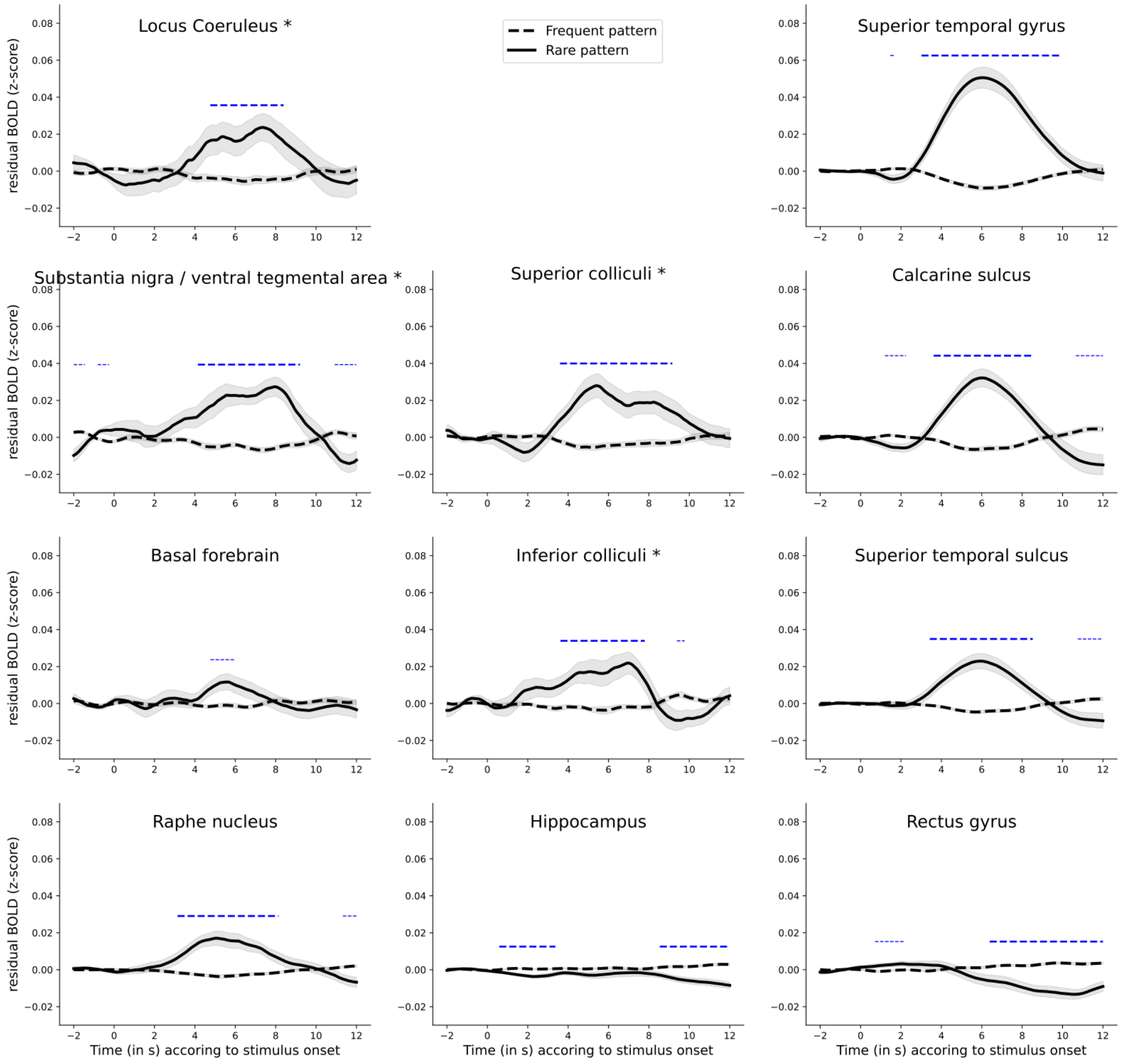

Figure S1. Time course of fMRI activity (z-score) evoked by rare patterns (bold line) and frequent patterns (dashed-line) in different brain structures. The first column shows neuromodulator nuclei, the second column other subcortical ROIs, and the third column cortical ROIs. Horizontal dashed blue lines correspond to clusters of significant difference ($p < 0.05$) and bold dashed blue lines to significant clusters after FWE correction ($p_{FWE} < 0.05$).

Table S2. Statistics for the effect of rare patterns in FIR analyses (max t-values, max p-values, max Cohen's d, and p-values for clusters with FWE correction), for all ROIs. When several clusters have been identified, the one with the higher max Cohen's d is reported. Note that for these analyses, the significant cluster identified for the LC did not reach significance when corrected for multiple comparisons across time.

Neuromodulation-related nuclei	Other subcortical nuclei	Cortical areas
**LC**<br>t_{max} = 4.11<br>p_{max} < 0.001<br>d_{max} = 0.84<br>cluster p_{FWE} < 0.001	**Superior colliculi**<br>t_{max} = 4.19<br>p_{max} < 0.001<br>d_{max} = 0.86<br>cluster p_{FWE} = 0.002	**Superior temporal gyrus**<br>t_{max} = 8.86<br>p_{max} = < 0.001<br>d_{max} = 1.82<br>cluster p_{FWE} = < 0.001
**SN/VTA**<br>t_{max} = 6.28<br>p_{max} < 0.001<br>d_{max} = 1.28<br>cluster p_{FWE} < 0.001	**Inferior colliculi**<br>t_{max} = 4.36<br>p_{max} < 0.001<br>d_{max} = 0.89<br>cluster p_{FWE} < 0.001	**Calcarine sulcus**<br>t_{max} = 5.75<br>p_{max} = < 0.001<br>d_{max} = 1.17<br>cluster p_{FWE} = <0 .001
**BF**<br>t_{max} = 4.72<br>p_{max} < 0.001<br>d_{max} = 0.94<br>cluster p_{FWE} < 0.001	**Hippocampus**<br>t_{max} = 2.27<br>p_{max} = 0.033<br>d_{max} = 0.46<br>cluster p_{FWE} = 0.277	**Superior temporal sulcus**<br>t_{max} = 5.74<br>p_{max} = < 0.001<br>d_{max} = 1.17<br>cluster p_{FWE} = < 0.001
**RN**<br>t_{max} = 5.03<br>p_{max} < 0.001<br>d_{max} = 1.03<br>cluster p_{FWE} < 0.001		**Rectus gyrus**<br>t_{max} = -7.83<br>p_{max} = < 0.001<br>d_{max} = -1.60<br>cluster p_{FWE} = < 0.001

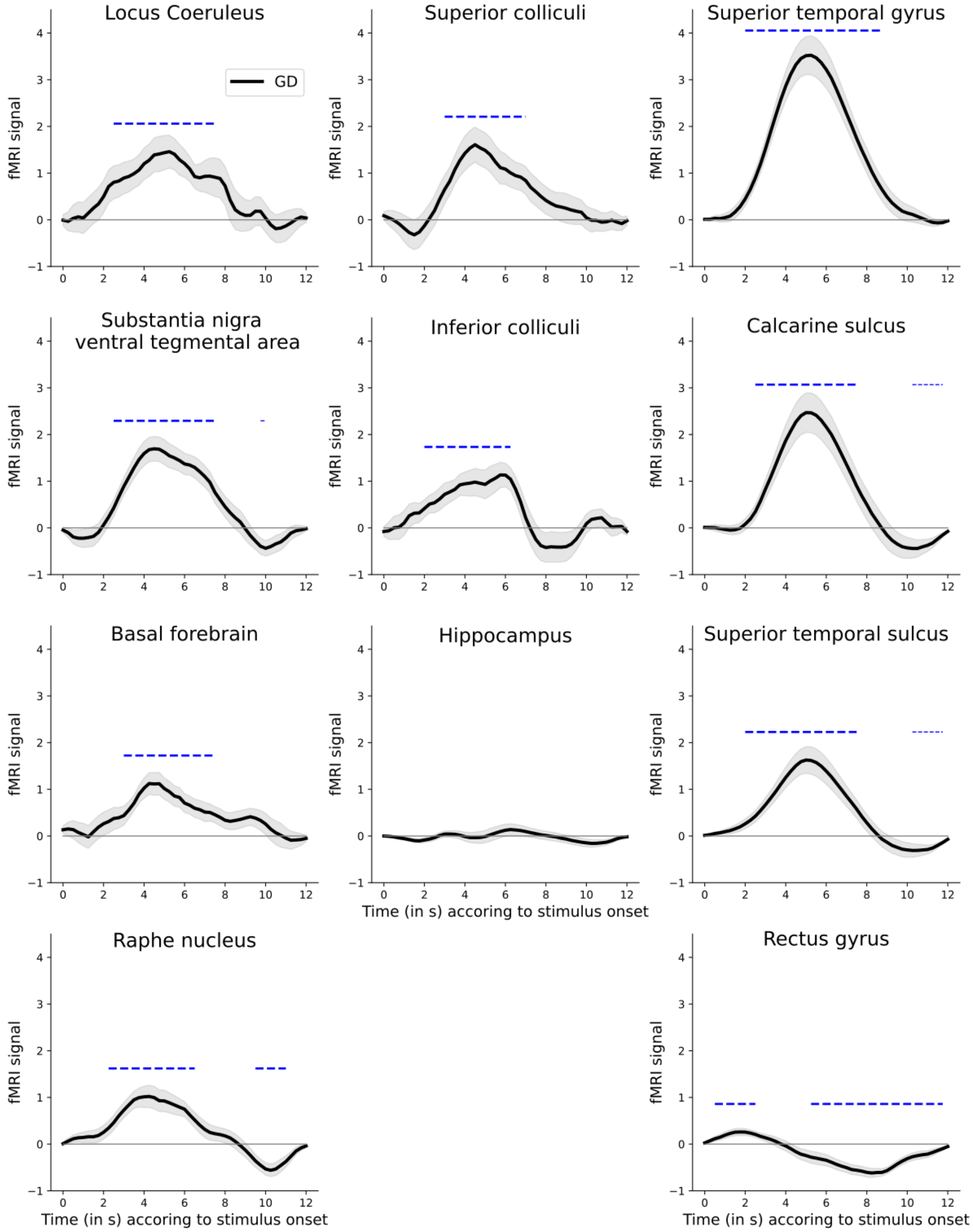

Figure S2. Time course of fMRI activity estimated using a FIR model for rare patterns. The first column shows neuromodulator nuclei, the second column other subcortical ROIs, and the third column cortical ROIs. Error shading is standard error. Blue dashed lines indicate significant clusters different from 0 (p_{FWE}<0.05).

Table S3. Statistics for the difference between the effects of rare and frequent patterns in GLM analyses (t-values, p-values, and Cohen's d), for all ROIs.

Neuromodulation-related nuclei	Other subcortical nuclei	Cortical areas
**LC**<br>$t(23) = 4.59$<br>$p < 0.001$<br>$d = 0.94$	**Superior colliculi**<br>$t(19) = 5.52$<br>$p < 0.001$<br>$d = 1.13$	**Superior temporal gyrus**<br>$t(23) = 9.20$<br>$p < 0.001$<br>$d = 1.88$
**SN/VTA**<br>$t(23) = 5.44$<br>$p < 0.001$<br>$d = 1.11$	**Inferior colliculi**<br>$t(23) = 2.53$<br>$p = 0.019$<br>$d = 0.52$	**Calcarine sulcus**<br>$t(23) = 6.51$<br>$p < 0.001$<br>$d = 1.33$
**BF**<br>$t(23) = 3.98$<br>$p = 0.001$<br>$d = 0.81$	**Hippocampus**<br>$t(23) = 1.50$<br>$p = 0.148$<br>$d = 0.31$	**Superior temporal sulcus**<br>$t(19) = 5.46$<br>$p < 0.001$<br>$d = 1.11$
**RN**<br>$t(19) = 4.41$<br>$p < 0.001$<br>$d = 0.90$		**Rectus gyrus**<br>$t(23) = -3.29$<br>$p = 0.003$<br>$d = -0.67$

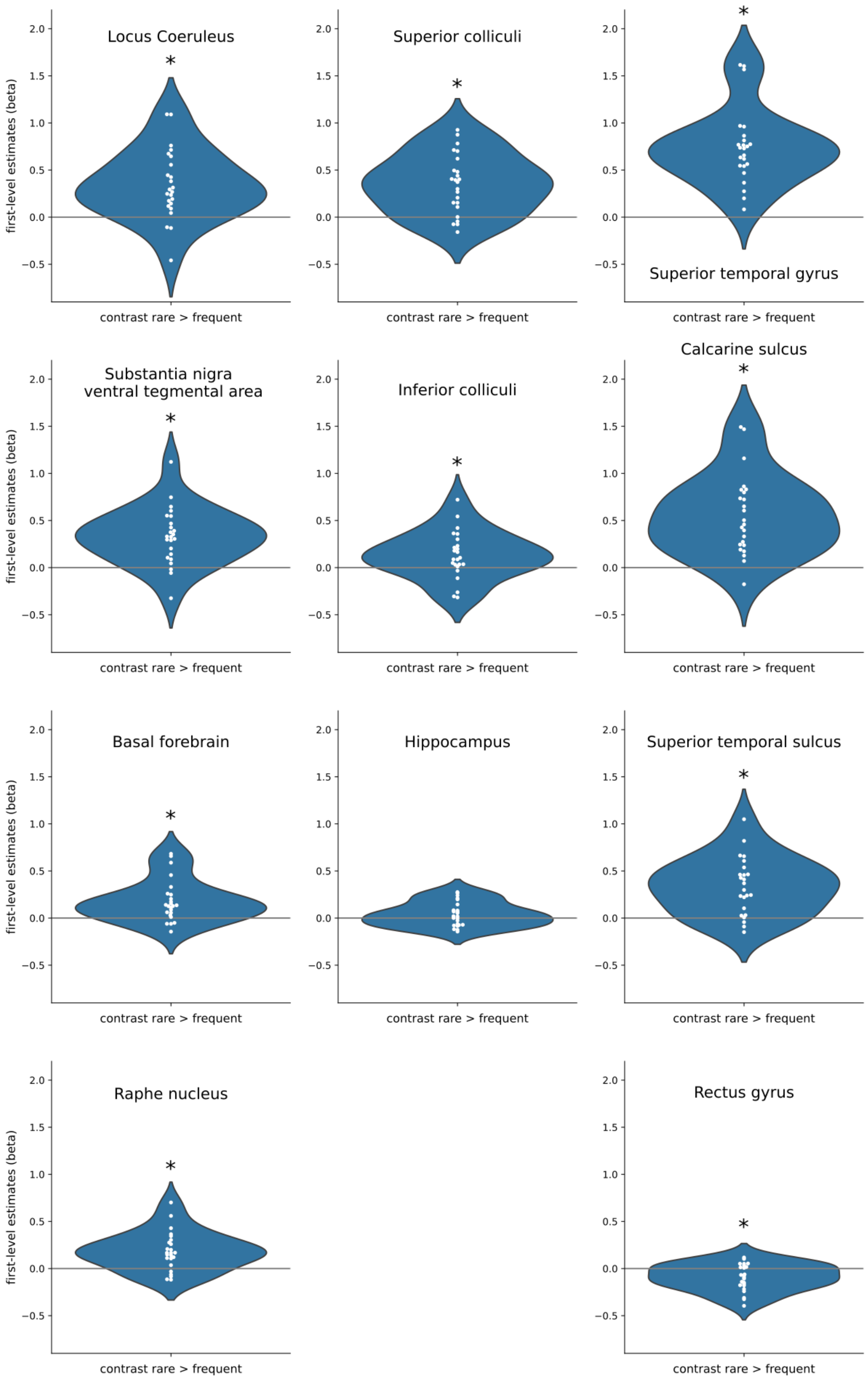

Figure S3. Single-subject estimates for the effect of rare patterns in different brain structures. The first column shows neuromodulator nuclei, the second column other subcortical ROIs, and the third column cortical ROIs. Stars indicate group-level significance ($p < 0.05$).

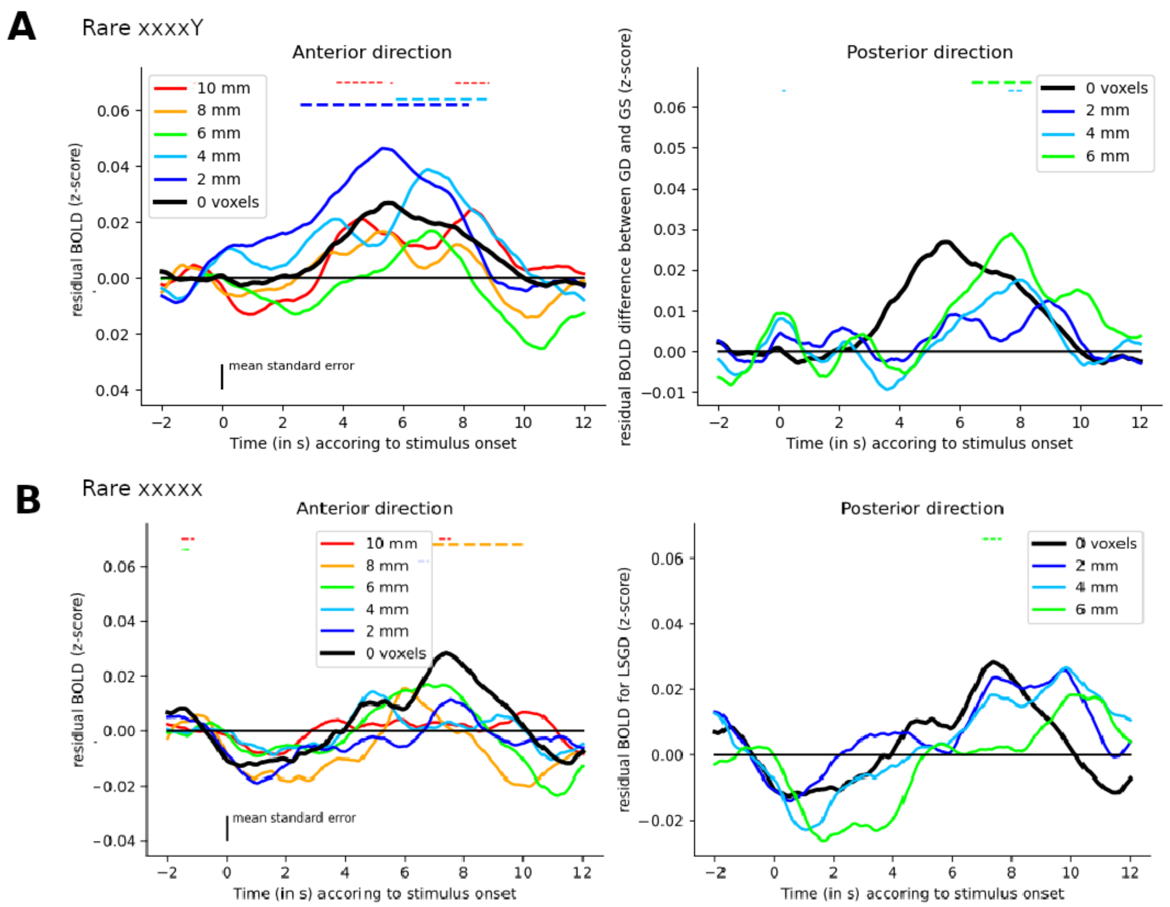

Figure S4. Time course of the z-scored fMRI activity for the two types of deviants or different shifts of the anatomically-defined LC ROI in millimeters. Two mm corresponds to 1 voxel. Colored horizontal dashed lines indicate the significant clusters ($p<0.05$; FWE corrected) identified for the difference between the corresponding color and the black line. A) For the rare xxxxY stimulus. Left panel refers to shifts toward the front of the head. Right panel refers to shifts toward the back of the head (see Figure 4B). Black line refers to the original, non-shifted LC ROI. B) For the rare xxxxx stimulus.

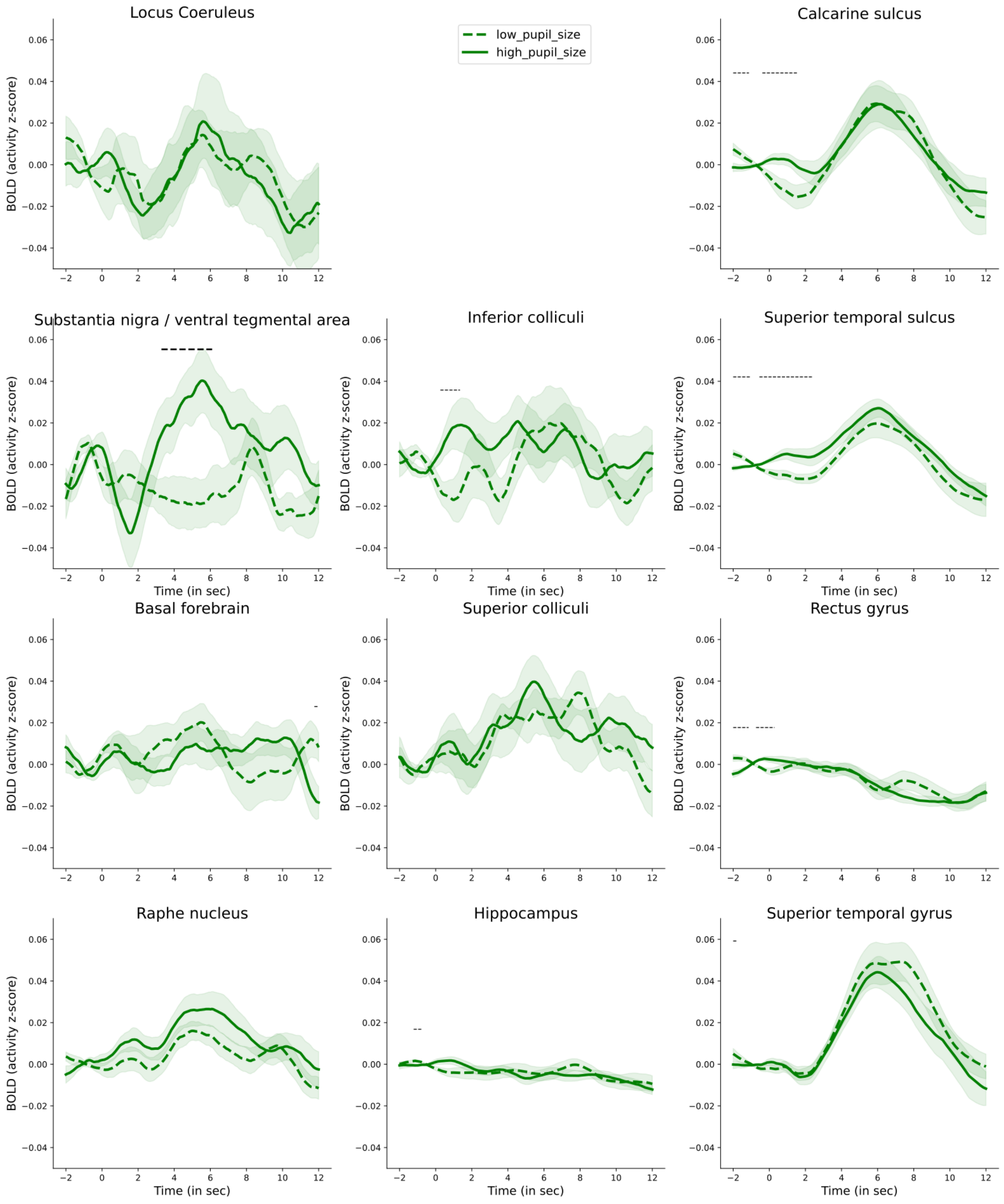

Figure S5. Time course of the effect of global deviance (z-scored fMRI activity elicited by rare), sorted by pupil response (low pupil size and large pupil size; median split), in all subcortical ROIs. Horizontal dashed black lines correspond to clusters of significant difference ($p < 0.05$). Bold dashed black lines correspond to clusters of significant difference after FWE correction ($p < 0.05$). N = 13. Mean number of included trials = 67.6 (min=29, max=88). The fMRI signal followed our prediction only in the SN/VTA ($t_{max} = 4.05$, $p_{max} = 0.002$, $d_{max} = 1.12$, cluster $p_{FWE} = 0.022$). No significant difference was found in the LC.

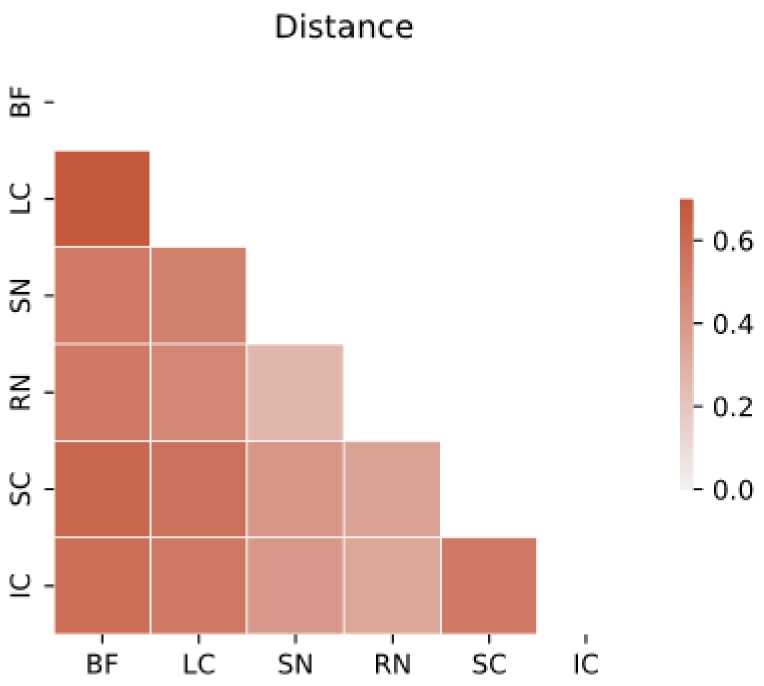

Figure S6. Distance matrix (used to make the dendrogram presented Figure 6B in the manuscript) characterizing the patterns of intrinsic correlations between each subcortical region and all cortical regions (covered in the field of view), computed for all pairs of subcortical regions. A larger distance between two regions indicates a greater difference in their patterns of intrinsic correlations with cortical regions.

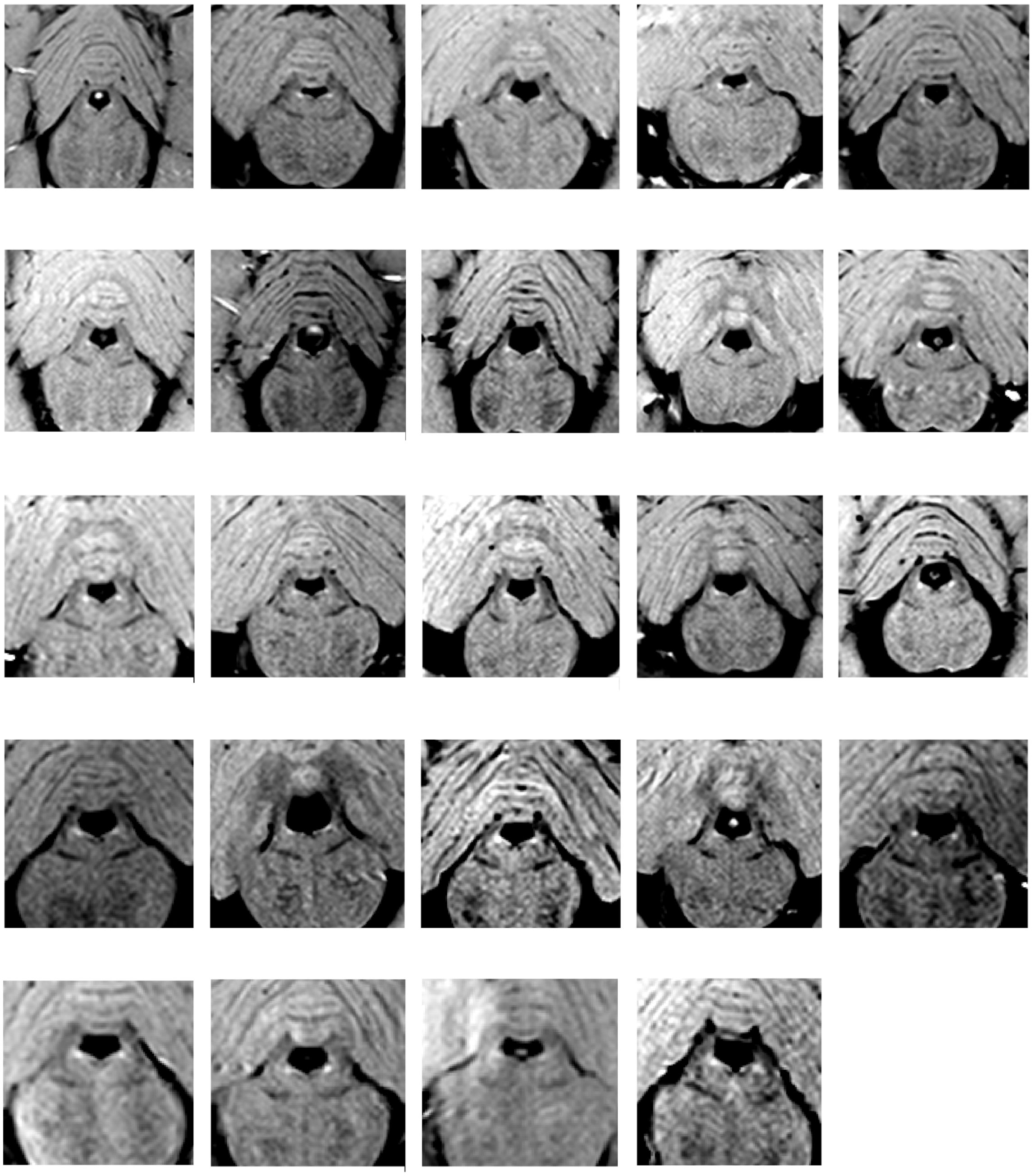

Figure S7. Anatomical images showing the LC in hypersignal for each participant.

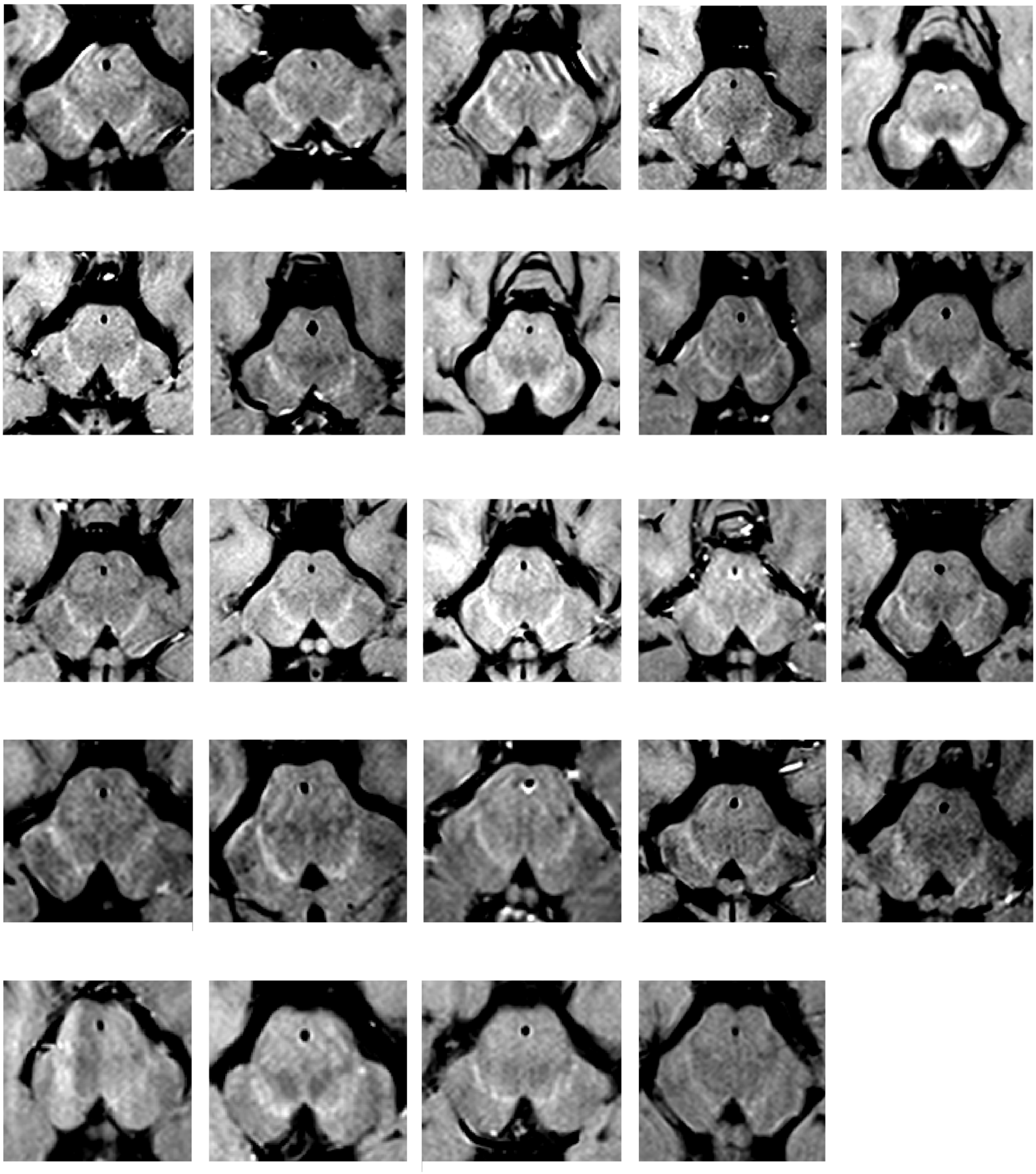

Figure S8. Anatomical images showing the SN/TVA in hypersignal for each participant.
