# Supplementary material for: Brainstem fMRI signaling of surprise across different types of deviant stimuli
Source: Cell Rep. 2023 Nov 10;42(11):113405. doi: 10.1016/j.celrep.2023.113405 (PMC10698303; doi:10.1016/j.celrep.2023.113405)
Supplement: Document S2. Article plus supplemental information [file mmc2.pdf]

Cell Reports

Article

Brainstem fMRI signaling of surprise across different types of deviant stimuli

Graphical abstract

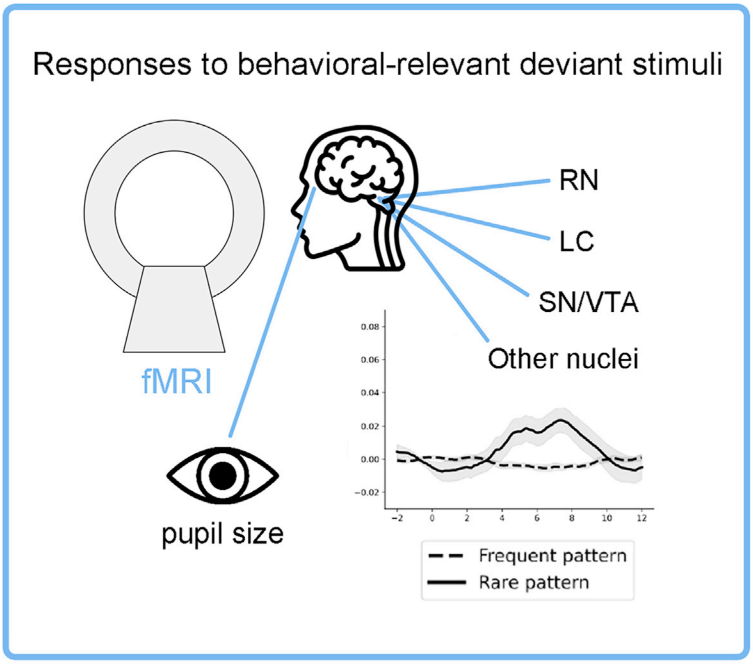

Highlights

- fMRI responses to task-relevant deviant stimuli are distributed in subcortical structures
- Similar responses are observed in pupil size, cortex, and subcortical structures
- Subcortical structures respond similarly to computationally different types of deviance

Authors

Audrey Mazancieux, Franck Mauconduit, Alexis Amadon, Jan Willem de Gee, Tobias H. Donner, Florent Meyniel

Correspondence

audrey.mazancieux@gmail.com (A.M.),
florent.meyniel@cea.fr (F.M.)

In brief

Mazancieux et al. use fMRI methods optimized for the brainstem and pupillometry to map deviant-related responses in subcortical structures in humans. They show that the detection of task-relevant deviant items recruits the same subcortical systems across computationally different types of deviance. This system involves the LC and other neuromodulatory systems.

Mazancieux et al., 2023, Cell Reports *42*, 113405
November 28, 2023 © 2023 The Author(s).
https://doi.org/10.1016/j.celrep.2023.113405

CellPress

Cell Reports

CellPress
OPEN ACCESS

Article

Brainstem fMRI signaling of surprise across different types of deviant stimuli

Audrey Mazancieux,[1,*] Franck Mauconduit,[2] Alexis Amadon,[2] Jan Willem de Gee,[3] Tobias H. Donner,[4] and Florent Meyniel[1,5,6,*]

[1]Cognitive Neuroimaging Unit, Institut National de la Santé et de la Recherche Médicale, Commissariat à l'Energie Atomique et aux énergies alternatives, Centre national de la recherche scientifique, Université Paris-Saclay, NeuroSpin center, 91191 Gif/Yvette, France
[2]NeuroSpin, CEA, CNRS, BAOBAB, Université Paris-Saclay, Gif-Sur-Yvette, France
[3]Department of Neuroscience, Baylor College of Medicine, Houston, TX, USA
[4]Section Computational Cognitive Neuroscience, Department of Neurophysiology and Pathophysiology, University Medical Center Hamburg-Eppendorf, Hamburg, Germany
[5]Institut de neuromodulation, GHU Paris, psychiatrie et neurosciences, centre hospitalier Sainte-Anne, pôle hospitalo-universitaire 15, Université Paris Cité, Paris, France
[6]Lead contact
*Correspondence: audrey.mazancieux@gmail.com (A.M.), florent.meyniel@cea.fr (F.M.)
https://doi.org/10.1016/j.celrep.2023.113405

SUMMARY

Detection of deviant stimuli is crucial to orient and adapt our behavior. Previous work shows that deviant stimuli elicit phasic activation of the locus coeruleus (LC), which releases noradrenaline and controls central arousal. However, it is unclear whether the detection of behaviorally relevant deviant stimuli selectively triggers LC responses or other neuromodulatory systems (dopamine, serotonin, and acetylcholine). We combine human functional MRI (fMRI) recordings optimized for brainstem imaging with pupillometry to perform a mapping of deviant-related responses in subcortical structures. Participants have to detect deviant items in a "local-global" paradigm that distinguishes between deviance based on the stimulus probability and the sequence structure. fMRI responses to deviant stimuli are distributed in many cortical areas. Both types of deviance elicit responses in the pupil, LC, and other neuromodulatory systems. Our results reveal that the detection of task-relevant deviant items recruits the same multiple subcortical systems across computationally different types of deviance.

INTRODUCTION

Detecting deviant stimuli (i.e., stimuli that violate some regularity) is crucial in a variety of processes, such as learning under uncertainty,[1] interacting in a flexible manner with the environment,[2,3] and orienting behavior.[4] In terms of mechanisms, it seems clearly established that deviance detection triggers the phasic, brain-wide release of noradrenaline from the locus coeruleus (LC) located in the brainstem,[4–8] especially when deviant items are behaviorally relevant and correctly detected.[9] This conclusion is supported mostly by studies in non-human animals, using electrophysiological recordings of LC neurons during oddball tasks in which frequent and rare stimuli are typically presented in a sequence: the LC responds specifically to the rare (hence, deviant) stimulus.[5,9–12] Studies in humans provided converging evidence: the functional MRI (fMRI) signal in the LC region increased after deviant stimuli in oddball tasks.[13,14]

However, this body of work leaves unclear the anatomical specificity of deviant-related responses: are they specific to the LC or shared across multiple other subcortical structures, notably neuromodulatory centers? The latter seems likely because deviance detection overlaps with other notions such as novelty[15,16] and unexpectedness[17,18] when deviance is defined by rareness, and salience[19] is known to implicate noradrenaline but also other neuromodulators.[20–28] For instance, dopamine encodes unexpected stimuli in the form of reward prediction error[24,25] as well as salient stimuli related to novelty.[20,22,28]

Several pharmacological studies indicate that the deviance-related response recruits a large set of neuromodulatory systems. Propranolol (a blocker of the noradrenergic β receptors) decreased fMRI signals in cortical regions that respond to deviant stimuli,[29] but this effect is not specific to noradrenaline. For instance, the P300 event-related potential (ERP) was used as an indicator of LC activity supported by photoactivation studies in rats,[27] and it was found to be larger for deviant stimuli.[30] However, this deviant-related P300 response was also found to be reduced following the administration of either scopolamine (a cholinergic antagonist) in rats[31] and humans[23] or clonidine (a noradrenaline α-receptor agonist) in humans.[21]

Pupillometry has also often been used as an indirect marker of LC activity, and the existence of pupil responses to deviant stimuli is clearly established.[32–35] However, a change in pupil size is not necessarily due to a change in LC activity[36] because other subcortical nuclei such as the inferior colliculi[37,38] and neuromodulators are at play such as acetylcholine from the basal forebrain,[39] and, more indirectly, serotonin from the raphe nucleus.[40]

This is an open access article under the CC BY license (http://creativecommons.org/licenses/by/4.0/).

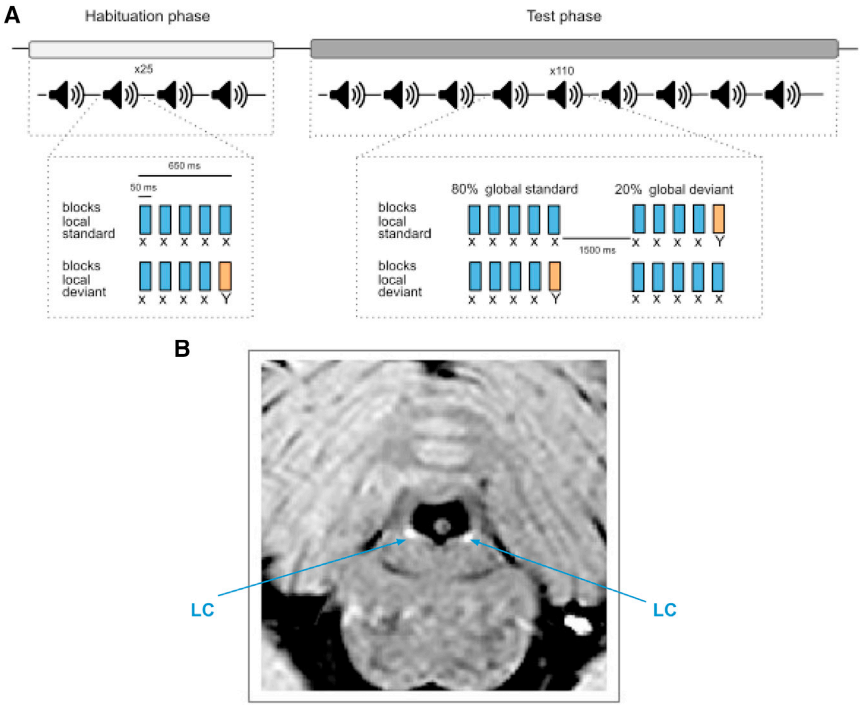

Figure 1. Task and example of anatomical images

(A) The local-global paradigm. Patterns correspond either to five identical tones ("local standards," xxxxx) or four identical tones and a different one ("local deviants," xxxxY). During the habituation phase, only one of the two patterns is presented (called the global standard pattern). During the test phase, this pattern is presented 80% of the time, and the other pattern (called the global deviant pattern) is presented in 20% of the cases. In total, participants are presented with four different types of patterns: when the global standard (80%) is the local standard (xxxxx), the global deviant (20%) is the local deviant (xxxxY); when the global standard (80%) is the local deviant (xxxxY), the global deviant (20%) is the local standard (xxxxx).

(B) Example slice of the anatomical turbo spin echo image used to delineate the LC (appearing in hypersignal, i.e., brighter) in each participant.

Here, we propose to measure deviant-related responses not only in the LC but also in other structures of the brainstem, notably in neuromodulatory centers. Direct, concurrent electrophysiological recording of multiple neuromodulatory centers is very difficult in non-human animals[41] and is not an option in humans. In contrast, fMRI can provide complete coverage of the brainstem (and beyond), but brainstem fMRI is challenging due to the presence of larger physiological noise compared to cortex and the very small size of the structures of interest, such as the LC.[42] We thus used fMRI methods optimized for the brainstem[13,38] and delineated the LC (noradrenaline), the substantia nigra/ventral tegmental area (SN/VTA; dopamine), and the superior and inferior colliculi (involved in pupil size and auditory processing[37,43,44]), based on the participant's anatomy. Most fMRI studies that measured LC activity used anatomical atlases, but this method is imprecise given its small size.[45] We also included for comparison the activity of other neuromodulatory regions: the basal forebrain (BF) for acetylcholine and the raphe nucleus (RN) for serotonin (using atlases because they are more difficult to delineate individually) as well as other subcortical and cortical areas.

The studies mentioned so far used oddball (or similar) tasks,[5,9–11,13,14,29] in which deviant (oddball) items typically differ greatly from the standard items in terms of physical properties (e.g., pitch difference) and probability, making them very salient. The deviant-related response may thus not reflect the detection of the deviant item per se, but downstream processes related to the salience or behavioral relevance of the deviant item. Here, we used the local-global paradigm[46] in which participants counted deviant items, making these deviant items behaviorally relevant (hence, salient). Interestingly, this paradigm differentiates between two types of deviant items: one based on the stimulus probability (just as in classical oddball tasks), which can be detected by simple mechanisms such as stimulus-specific adaptation[43,44]; and another based on the structure of the sequence, which requires more elaborate processes to be detected[47] such as predictive coding[48] and even awareness.[49,50] In other words, this paradigm dissociates the notion of task-relevant deviant items from a specific type of deviance. We investigated whether the responses to these task-relevant deviant items are similar or different between these two types of deviant items.

To anticipate our result, we found that both types of deviant items elicit widespread fMRI responses in subcortical and cortical structures, which may correspond to the broadcasting of a task-related, salient event downstream of potentially different deviance detection systems.

RESULTS

Distinguishing between different types of deviant items

The local-global paradigm presents sequences of stimuli that exhibit two nested levels of structure. At the local level, stimuli form patterns of four sounds with a fifth one that is either identical, forming a locally standard pattern xxxxx, or different, forming a locally deviant pattern xxxxY (Figure 1A). The global level is characterized by the succession of patterns separated by short pauses: in each block of the task, one pattern is frequent (80% of patterns) while the other is rare (20%). The local and global properties, as well as sound identity, were crossed in a full factorial 2 × 2 × 2 design. In each block, participants were first familiarized with the frequent pattern only (which defines the block identity: xxxxx block or xxxxY block) and then presented with a few rare patterns (called global deviants) interleaved among the frequent ones. Participants listened to these patterns and were instructed to count the number of global deviant patterns. Twenty participants performed the task in the scanner where they had to count the number of rare patterns for a total of four sessions.

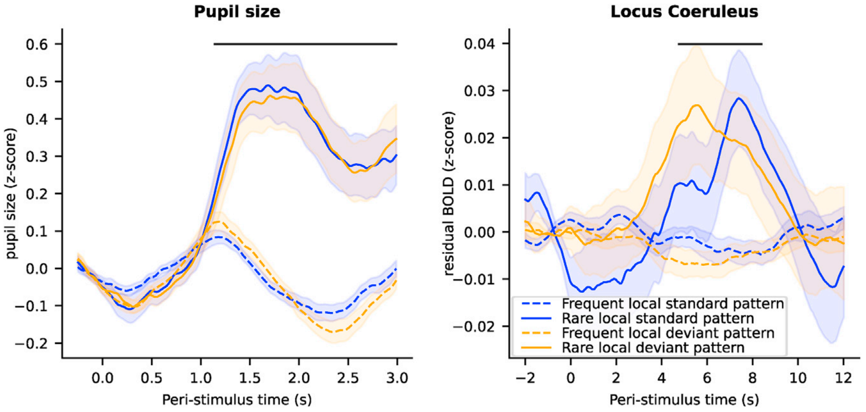

Figure 2. Epoch-based analyses of the four stimuli for pupil and LC data

(A) Pupil size (*Z* score) evoked by the four types of patterns.

(B) Time course of fMRI activity (*Z* score) in the LC evoked by the four types of pattern. Error shading is standard error (n = 24). Black dashed lines indicate significant clusters for the effect of rare patterns (non-parametric cluster-level paired t test for global effect, $p_{FWE} < 0.05$).

Note that there is a key difference between the two types of global deviants. A rare xxxxY can be detected among frequent xxxxx based on low-level processes that operate on the probability of the sounds themselves in a sequence, like stimulus-specific adaptation, because the Y sound is extremely rare (occurring only with probability $0.2 \times 1/5 = 0.04$). In contrast, such low-level processes do not suffice to detect a rare xxxxx among frequent xxxxY because with x being the dominant sound (occurring with probability $0.8 \times 1 + 0.2 \times 4/5 = 0.96$), xxxxY stands out more than xxxxx based on sound probability alone. A mechanism for the detection of a rare xxxxx must operate at a higher level, namely the sequence of sound patterns. The local-global paradigm thus allows a distinction between different computations for deviance detection, operating on the stimulus probability and sequence structure, respectively, and possibly different mechanisms, such as bottom-up and top-down processes (see discussion).

Robust responses to global deviants in the pupil-linked arousal system

Pupil size is controlled by the autonomic nervous system. It provides a marker of arousal that is known to transiently increase when deviant stimuli are detected.[51] To characterize its response to global deviance, we performed a baseline-corrected, epoch-based analysis to isolate the phasic evoked response (see STAR Methods). This analysis included only a subset of 13 participants who have a large-enough number of trials after artifact rejection (see STAR Methods). Pupil size exhibited a clear response to global deviants, with larger pupil size for rare patterns compared to frequent patterns (maximum t value [t_{max}] = 6.85, maximum p value [p_{max}] < 0.001, maximum Cohen's d [d_{max}] = 1.90, p value for clusters with family-wise error [cluster p_{FWE}] < 0.001) (Figure 2A). Note that the response was similar between the two types of global deviants (there was no significant effect of local deviance, $t_{max} = 1.95$, $p_{max} = 0.075$; or interaction between local and global deviances, $t_{max} = 1.35$, $p_{max} = 0.201$).

Global deviance transiently increases LC activity

Having established that both types of global deviants in the local-global paradigm elicit strong transient activation of the pupil-linked arousal system, we next investigated their effects in our primary region of interest (ROI), namely the LC. As for the pupil, we used epoch-based analyses. We extracted time series from the LC, removed several confounds (see STAR Methods), epoched the signal (from −2 to 12 s around the onset of patterns), and corrected for the baseline (subtracting the signal from −2 to 0 s). This analysis allowed us to track the fMRI activity of the LC in response to deviant stimuli with no assumption about the shape of the hemodynamic response, which may be different in subcortical structures compared to the cortex where the canonical hemodynamic responses have been defined.[52] In addition, as for the pupil size analyses, the baseline correction removes the autocorrelation that may exist in the signal before and after the pattern onset and captures the change in activity evoked by the pattern. Thus, this analysis captures the phasic activity of the LC and removes the tonic activity.

The LC activity showed a main effect of global deviance ($t_{max} = 3.04$, $p_{max} = 0.006$, $d_{max} = 0.62$, cluster $p_{FWE} = 0.024$) with a greater increase in fMRI activity for rare patterns compared to frequent patterns (Figure 2B). No cluster was identified for either the local effect or for the interaction between the local and global deviance, suggesting that responses were similar between the two types of global deviants.

Mapping of deviance-related responses across regions of interest

We repeated the epoch-based analysis to quantify deviant-related responses in several other brain structures sorted into three categories. (1) The other neuromodulator nuclei included the SN/VTA (individually delineated, see STAR Methods and Figure S5), the BF, and the RN (based on anatomical atlases). (2) The other subcortical structures included the superior colliculus (SC; involved in orienting responses), the inferior colliculus (IC; involved in auditory processing), and the hippocampus (involved in sequence processing). (3) Cortical structures, where the fMRI signal-to-noise ratio is higher than in subcortical structures, included the superior temporal gyrus (previously identified to respond to rare patterns with the same task, see Bekinschtein et al.[46]), the primary auditory and visual cortices (corresponding to the calcarine and the superior temporal sulcus in Destrieux's parcellation[53]), and the rectus gyrus in the medial prefrontal cortex (which is part of the default mode network and thus not expected to respond to rare patterns (see Figure S1 for the effect of rare patterns in a full parcellation of the brain using Destrieux's parcellation).

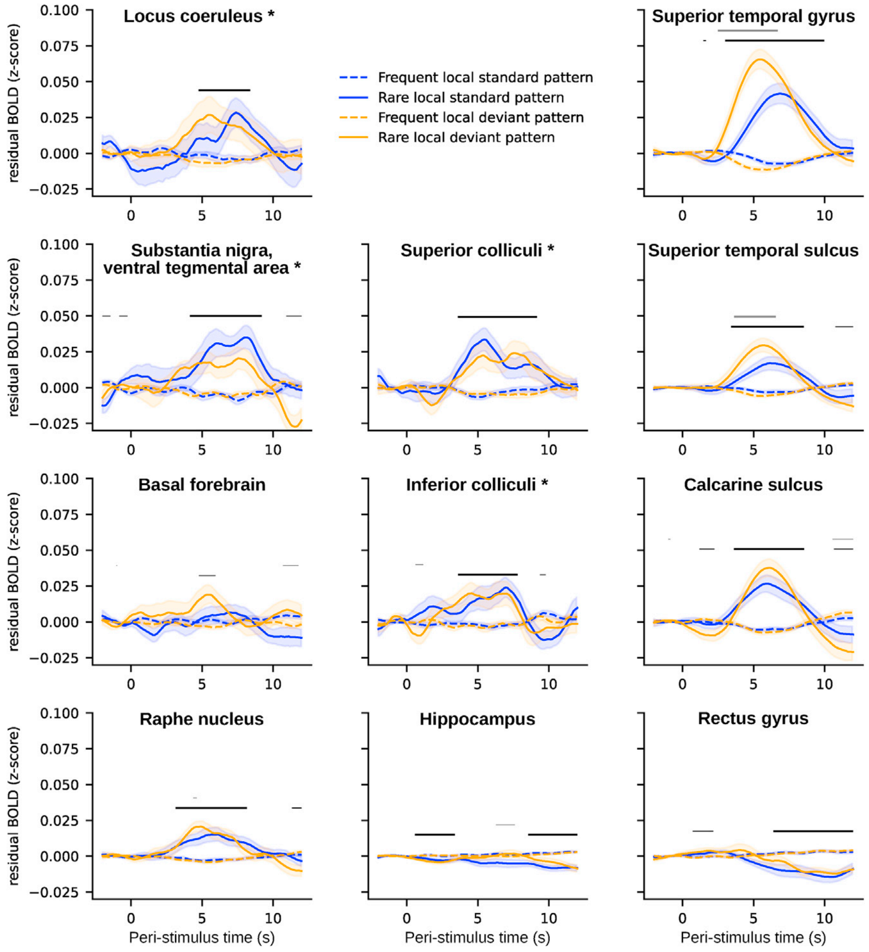

Figure 3. Time course of fMRI activity (*Z* score) evoked by the four types of patterns

The first column shows neuromodulator nuclei, the second column other subcortical regions of interest (ROIs), and the third column cortical ROIs. Asterisks indicate ROI defined in native space by manual delineation. Error shading is standard error (n = 24). Bold black lines indicate significant clusters for the effect of rare patterns (global effect), and bold gray lines indicate clusters for the interaction between the global effect and the local effect (non-parametric cluster-level paired t test, $p_{FWE} < 0.05$). Non-bold lines indicate time points with significant differences (non-parametric cluster-level paired t test, $p < 0.05$ but cluster $p_{FWE} > 0.05$).

Figure 3 shows the four types of stimuli for each ROI, and Table 1 summarizes the corresponding statistics (see Figure S2 for a similar figure gathering the two types of rare and frequent patterns). All neuromodulator nuclei showed an increase in fMRI activity for rare patterns compared to frequent patterns (which remained significant when corrected for multiple comparisons across time, except in the BF). The dopaminergic SN/VTA is the structure that elicits the largest response. For the LC, the maximum effect size was smaller than in the SN/VTA and the RN, probably due to the smaller size of the LC ROI. The ventral medial prefrontal cortex and the hippocampus showed the reverse pattern later in the time window: fMRI activity in these regions was higher for frequent stimuli compared to rare stimuli. The other structures (cortical and subcortical) all exhibited a larger response for rare patterns compared to frequent patterns. Larger responses were found in cortical ROIs than in subcortical areas, potentially due to their different sizes and signal-to-noise ratios (SNRs). The shape of the response also differed between cortical and subcortical regions: cortical regions had a more canonical response with a clear peak while several subcortical regions exhibited a kind of plateau (Figure 3). Note that caution should prevail when comparing these different structures. First, cortical and subcortical structures differ in terms of SNR. Second, within subcortical structures, four have been defined in native space and others using atlases render the latest less neuroanatomically accurate.

No ROI showed a main effect for local deviance. Interaction between the local and global effect was significant only for cortical areas involved in auditory processing, namely the superior temporal gyrus ($t_{max} = 6.08$, $p_{max} < 0.001$, $d_{max} = 1.24$, cluster $p_{FWE} < 0.001$) and the superior temporal sulcus ($t_{max} = 3.84$, $p_{max} < 0.001$, $d_{max} = 0.78$, cluster $p_{FWE} = 0.045$). In these regions, the signal time courses (Figure 3) indicate that the interaction originates from the fact that the rare xxxxY patterns (the stimulus-probability deviant) elicited a higher and earlier response than the rare xxxxx patterns (the structure deviant). To confirm this hypothesis, we performed a follow-up analysis to estimate the difference in response peaks between conditions. The superior temporal gyrus showed a significant difference in the peak of the response, which was significantly earlier for rare xxxxY than rare xxxxx patterns (estimated difference 1.422 s; 95% confidence interval [CI] 0.888, 2.004). A similar trend was observed in the superior temporal sulcus (mean 0.672, 95% CI −0.102, 1.742).

To rule out that the detection of global deviant-related responses depends on the specifics of our analysis approach, we compared frequent and rare patterns across brain structures using finite impulse response (FIR) analyses and general linear model (GLM) analyses (see supplemental information). Epoch-based analyses do not model the potential superposition of effects of the current and previous patterns in the time window of interest. In contrast, FIR and GLM analyses are designed to model this superposition and differ in their assumptions about the hemodynamic response (which is unconstrained or assumed to be canonical, respectively). The epoch-based analysis also contains a baseline correction that aims to suppress endogenous fluctuations in the signal—therefore focusing on phasic activity—which are ignored by FIR and GLM analyses. These three analyses are thus complementary.

Table 1. Statistics for the global effect for all ROIs

Neuromodulation-related nuclei	Other subcortical nuclei	Cortical areas
LC	superior colliculi	superior temporal gyrus
$t_{max} = 3.04$	$t_{max} = 4.24$	$t_{max} = 8.52$
$p_{max} = 0.006$	$p_{max} < 0.001$	$p_{max} < 0.001$
$d_{max} = 0.62$	$d_{max} = 0.86$	$d_{max} = 1.74$
cluster $p_{FWE} = 0.023$	cluster $p_{FWE} = 0.005$	cluster $p_{FWE} < 0.001$
SN/VTA	inferior colliculi	calcarine sulcus
$t_{max} = 5.52$	$t_{max} = 3.46$	$t_{max} = 6.31$
$p_{max} < 0.001$	$p_{max} = 0.002$	$p_{max} < 0.001$
$d_{max} = 1.13$	$d_{max} = 0.71$	$d_{max} = 1.29$
cluster $p_{FWE} < 0.001$	cluster $p_{FWE} = 0.004$	cluster $p_{FWE} < 0.001$
BF	hippocampus	superior temporal sulcus
$t_{max} = 2.35$	$t_{max} = -4.69$	$t_{max} = 5.53$
$p_{max} = 0.027$	$p_{max} < 0.001$	$p_{max} < 0.001$
$d_{max} = 0.48$	$d_{max} = -0.96$	$d_{max} = 1.12$
cluster $p_{FWE} = 0.159$	cluster $p_{FWE} = 0.022$	cluster $p_{FWE} < 0.001$
RN	–	rectus gyrus
$t_{max} = 4.70$	–	$t_{max} = -5.05$
$p_{max} < 0.001$	–	$p_{max} < 0.001$
$d_{max} = 0.96$	–	$d_{max} = -1.03$
cluster $p_{FWE} = 0.002$	–	cluster $p_{FWE} = 0.006$

Statistical measures include non-parametric cluster-level paired t-tests, maximum t values, maximum p values, maximum Cohen's d, and p values for clusters with FWE correction.

Overall, results were qualitatively consistent across the three types of analyses, suggesting that the global effect does not depend on the type of analyses we performed. The only notable qualitative differences concern the hippocampus and the ventral medial prefrontal cortex where the late negative effect of global deviance found in epoch-based analyses (and FIR analyses) differed from the positive (non-significant) effect found with GLM analyses, probably because the late difference is not well captured by the canonical response function.

Anatomical specificity of the response to global deviants around the LC region

The global deviant-related response is very much distributed in cortical and subcortical structures, raising the concern that the effect found in the LC may not be specific to this region but instead may be widespread within the pons. To test for the anatomical specificity of global deviance within the pons, we repeated the same epoch-based analyses but after shifting the ROI corresponding to the LC in space. In the native space of each participants, we shifted this ROI toward the front of the head (from 1 to 5 voxels, i.e., +2 to +10 mm leading to five new ROIs, see Figure 4B) and toward the back of the head, which falls in the fourth ventricle (from 1 to 3 voxels, i.e., −2 to −6 mm, leading to three new ROIs, see Figure 4B). This axis is more relevant for the shift of the LC ROI than shifting toward left or right because the LC is a bilateral structure, and more relevant than toward the spinal cord or the midbrain because the LC is elongated along this axis. For each shift of the ROI, we extracted the corresponding time series and performed epoch-based analysis for global deviant and global standards stimuli.

Figure 4A shows the time course of the difference in fMRI signals between rare patterns and frequent patterns for different shifts of the LC ROI (see Figure S5 for the same analysis for the xxxxY pattern and the rare xxxxx pattern separately). The effect of global deviance overall decreased as larger shifts were applied: the maximum difference between rare and frequent pattern changed from 0.057 (SD = 0.020) originally to 0.051 (SD = 0.020), 0.051 (SD = 0.022), 0.042 (SD = 0.021), 0.031 (SD = 0.019), and 0.032 (SD = 0.020) in the anterior direction (+2 mm to +10 mm shifts) and to 0.041 (SD = 0.022), 0.043 (SD = 0.018), and 0.036 (SD = 0.020) in the posterior direction (−2 mm to −6 mm shift). Only the signal for the actual LC (no shift) and the signal for the +2 mm and −4 mm shift showed a significant effect of global deviance (no shift: p_{FWE} = 0.023; +2 mm shift: p_{FWE} = 0.006; −4 mm shift: p_{FWE} = 0.010). Direct comparisons of the global deviance in shifted and unshifted data showed time points with significant differences ($p < 0.05$, one-sided test, for shifts of +8 and −6 mm, but the corresponding cluster p_{FWE} remained >0.05).

We also performed GLM statistical mapping analysis for the effect of global deviant stimuli. Significant voxels in the brainstem showed some overlap with the LC atlas (Figure 4C). These results support an anatomical specificity of the effect of global deviance in the LC region compared to its vicinity, but without sharp boundaries.

Comparison of LC activity in native space and atlas

The anatomical specificity of the effect of global deviance in the LC region can also be assessed by comparing the results obtained with anatomical delineation of the LC in each subject (in native space) to the expectedly less accurate ones obtained from a probabilistic atlas of the LC (in standardized space, see Figure 5). We extracted fMRI time series based on an atlas of the LC (see STAR Methods) that identified 10 voxels in standardized space. Note that our delineation in native space identified only a mean of 5.54 voxels for the LC (minimum = 4 voxels, maximum = 9 voxels across participants). A comparison of the two approaches revealed that the voxels identified in native space were systematically in the more anterior part of the atlas of the LC and thus closer to the midbrain, which is consistent with previous studies.[55] Therefore, to allow a fair comparison with the individual delineations (Figure 2), we also performed a second analysis matched in voxel number (using 6 voxels in the atlas that were the most anterior, see Figure 5). The two atlas-based analyses showed significant effects of global deviance (full atlas: $t_{max} = 4.15$, $p_{max} < 0.001$, $d_{max} = 0.85$, cluster $p_{FWE} = 0.002$; atlas with 6 voxels: $t_{max} = 4.16$, $p_{max} < 0.001$, $d_{max} = 0.85$, cluster $p_{FWE} < 0.001$). No significant cluster was identified for the effect of local deviance, but the interaction between local deviance and global deviance was significant in both analyses (full atlas: $t_{max} = 4.15$, $p_{max} < 0.001$, $d_{max} = 0.85$, cluster $p_{FWE} = 0.025$; atlas with 6 voxels: $t_{max} = 2.93$, $p_{max} = 0.009$, $d_{max} = 0.66$, cluster $p_{FWE} = 0.022$). In contrast to the analysis

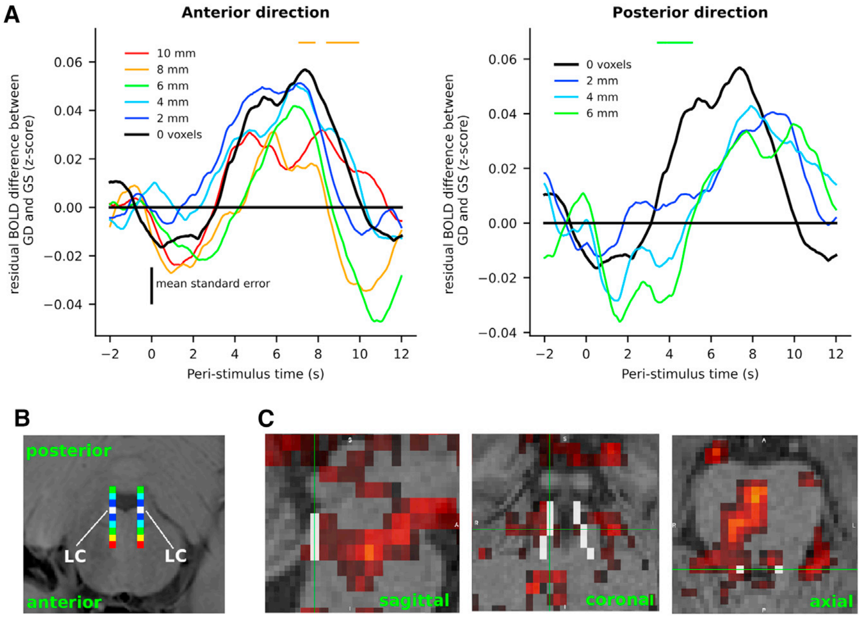

Figure 4. Anatomical specificity around the LC in the pons

(A) Time course of the effect of global deviance (difference in Z-scored fMRI activity between rare and frequent patterns) for different shifts of the anatomically defined LC ROI in millimeters (2 mm corresponds to 1 voxel). Left panel refers to shifts toward the anterior direction of the pons. Right panel refers to shifts toward the posterior direction of the pons. Black line refers to the original, non-shifted LC ROI. Colored horizontal dashed lines refer to identified clusters for the difference between the corresponding color and the black line. None of them remains significant after correction for multiple comparisons (non-parametric cluster-level paired t test for one-sided tests).

(B) Example location of the LC in the native space of one participant (in white) and the same ROI shifted along the anteroposterior axis (gradients from blue to red).

(C) Statistical Z-score map for the effect of global deviant stimuli, thresholded at $Z = 2.8$ (corresponding to $p = 0.005$). White voxels correspond to the LC atlas from Keren et al.[54] Mean standard error is mean standard error of all shifts ($n = 24$).

performed after individual delineation of the LC, a region corresponding to the atlas exhibits a response to global deviance that is mostly driven by stimulus probability (rare xxxxY patterns). This is in line with the fact that the response to the rare xxxxY is largely shared by voxels in the vicinity of the individually anatomically defined LC whereas the response to the rare xxxxx patterns is not, but instead is more specific to the LC itself (Figures S4A and S4B). If the atlas corresponds to voxels that are not exactly centered on the true individually and anatomically defined LC of each participant, but instead on its vicinity, the response to rare xxxxx patterns is expected to be reduced and even undetected.

Functional connectivity of subcortical regions

To explore how the different subcortical structures relate to one another, we performed a correlation analysis of intrinsic signals among subcortical regions. We used the residual time series (after removing the linear effect of external common causes—the stimuli—and potential confounds: movement parameters, signal in the 4th ventricle, and physiological signals) (Figure 6A). The LC signals correlated significantly with the other neuromodulatory centers (RN, SN/VTA; with the notable exception of the BF) and with the IC, which is known to be involved in auditory deviance detection.[43] SN/VTA and RN were the regions that correlated the most with other subcortical structures.

Correlations among subcortical structures are likely to be shaped by subcortical connections as well as afferent cortical connections. In a complementary analysis, we analyzed the pattern of correlations between cortical and subcortical signals. We measured the correlations of intrinsic signals between each subcortical structure and each cortical region that has substantial coverage in our field of view (>50% of the region, see supplemental information). We then used the cortical-subcortical correlation matrix to cluster hierarchically the subcortical structures (Figure 6B). The cortical correlation profile of the BF appeared markedly different from the other subcortical regions and formed a separated cluster. Within the second cluster, the two regions of the tectal system (SC and IC) and the three remaining neuromodulatory centers (LC, SN, and RN) were grouped together respectively. The subcortico-subcortical correlations (Figure 6) and the distance matrix between subcortical regions based on their cortical correlations (Figure 6) did not differ significantly between conditions (rare xxxxx blocs vs. rare xxxxY, all $p > 0.14$, except for the distance between the SC and the IC for which $p = 0.03$ without Bonferroni correction).

DISCUSSION

We performed a systematic response mapping in subcortical structures using fMRI coupled with pupillometry in a task that involves two types of deviants that requires computations based on the stimulus probability and sequence structure, respectively. Global deviance evoked transient LC responses, which was our primary ROI because it is well established that the central noradrenergic system vigorously responds to deviant stimuli.[5,9,10] Similar responses were found in other neuromodulatory centers (the SN/VTA and the RN), other subcortical nuclei (the SC and IC), and cortical regions (the anterior and posterior superior temporal gyri, the primary auditory and visual cortices). Correlations of intrinsic signals revealed the functional similarity of the RN, SN/VTA, and LC. Local and global deviances interacted in cortical responses related to auditory processing where global deviants elicited stronger and earlier responses when corresponding to a stimulus-probability deviant than a structure deviant. In contrast, subcortical structures (and the visual cortex) exhibited similar responses to both types of global deviants. This response to both types of global deviants showed some anatomical specificity to the LC region within the pons because it decreased when moving away from the subject-specific, anatomically

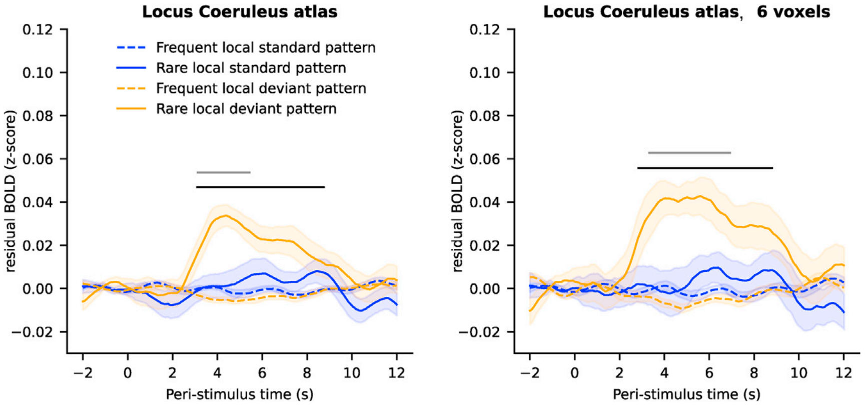

Figure 5. Anatomical specificity assessed with a normalized atlas of the LC

Time course of fMRI activity (*Z* score) in the LC evoked by the four types of patterns based on an anatomical atlas (left) and a selection of 6 voxels in the most anterior part of this atlas (right). Error shading is standard error (n = 24). Black and gray dashed lines indicate significant clusters for the global effect and the interaction between global and local effects, non-parametric cluster-level paired t test, respectively ($p_{FWE} < 0.05$).

defined LC region and became driven by stimulus-probability deviants when using a probabilistic, normalized atlas of the LC. Pupil size exhibited similar responses to both types of global deviants.

The two types of deviant items in the local-global paradigm act via different brain circuits, according to previous electrophysiological studies. Local deviance (xxxxY vs. xxxxx) elicits an early response in sensory cortices, whereas global deviance (rare vs. frequent patterns) elicits a later response that is distributed across brain areas and reaches the frontal lobe in both humans[46,56–58] and macaques.[59] The effects of local deviance and global deviance are propagated across cortical areas through different frequency bands, the gamma band and beta-alpha band, respectively[56,59] which are distinct functional markers of bottom-up and top-down processes.[60–62] Our results focused on a comparison of the two types of global deviance and showed that rare patterns elicited stronger and earlier responses when they corresponded to the xxxxY patterns (stimulus-probability deviant) than to the xxxxx pattern (structure deviant) in regions of the temporal lobe related to auditory processing, consistent with the idea that the detection of a rare xxxxx pattern recruits top-down processes (unfortunately, our brainstem-optimized partial brain coverage excluded most of the prefrontal cortex). We note that the distinction between global deviance based on stimulus probability and sequence structure is not tested in several previous studies,[46,51,56] leaving unclear whether the global effect analyzed in those studies is driven by both types of global deviants or just one.

In subcortical structures, in the pupil, and in the primary visual cortex, responses to both types of rare patterns were largely similar, in contrast to the temporal cortex. This similarity could indicate that they belong to a common final path, downstream of different circuits that detect different types of deviant items, which broadcasts the occurrence of a task-relevant, salient event. Subcortical structures such as the LC and the SN/VTA are known to be generally responsive to salient events, even when this salience is not due to the rareness of the event.[26–28,41] Determining the source and target of neural activity in those cortico-subcortical networks would be valuable but would require better time-resolved techniques than fMRI, such as electrophysiology, which is technically difficult to obtain. The LC in particular may play a central role in arousing the brain in response to task-relevant deviant stimuli. Studies in rodents and primates showed that afferent LC inputs mainly come from subcortical nuclei and that in cortex, only the prefrontal region directly projects to the LC.[63,64] Subcortical structures seem to be suitable candidates to signal the presence of rare xxxxY patterns but, as discussed above, they seem to lack the mechanisms to detect rare xxxxx patterns. The detection of the latter seems to rely on higher-order regions such as the prefrontal cortex, which could directly signal those types of deviants to the LC.

In the local-global task, the increase in central arousal that follows rare patterns depends on the participants' state of consciousness and their awareness of a sequence structure. Previous studies showed that the global deviance detection vanishes in patients with disorders of consciousness,[65] when healthy subjects fall asleep,[50] and when they are not aware of (or do not pay attention to) the task structure.[51] Interestingly, the effect of global deviance (notably rare xxxxx patterns) is more difficult to detect, and with a reduced extent, in brain recordings of macaque monkeys,[59,66–68] for which global deviants are not behaviorally relevant and thus potentially not attended, compared to healthy human participants who are told about the existence of global deviants and often asked to count them.[46,50,51,56,57] Here, we also asked participants to count the global deviants, which probably enhanced their detection and the associated brain responses.

It is important to note that our design cannot distinguish between deviance detection per se (e.g.,. expectation violation signal) and the consequences of deviance detection (e.g., the identification of a task-relevant event). In addition, our fMRI data do not indicate where the deviance detection is computed in the brain for the two types of deviants. We can only speculate that expectation violation in some regions may serve to detect deviant items, possibly based on different circuits for the two deviant types, and this detection may be communicated to (and enhanced by) some other regions due to its task relevance.

LC responses are known to depend on attentional effects. During active oddball tasks, LC neurons exhibited a higher response when monkeys correctly (vs. incorrectly) detected rare stimuli.[9] More generally, there are state-dependent changes in tonic LC activity: higher tonic activity coincides with periods when monkeys have more motor activity that is irrelevant to the task; in contrast, periods of drowsiness, immobility, and eye closure reduce LC activity.[9] Thus, phasic LC responses to

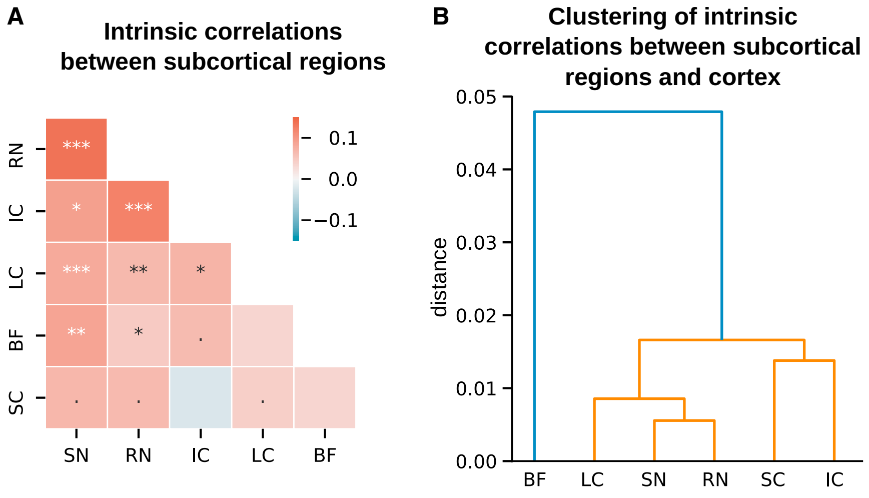

Figure 6. Functional connectivity of subcortical regions

(A) Correlation matrix of intrinsic signals across subcortical regions. Asterisks indicate Bonferroni-corrected significant Pearson correlations (*p < 0.05, **p < 0.005, ***p < 0.0005; a dot indicates p < 0.05 uncorrected).

(B) Hierarchical clustering of subcortical regions based on their patterns of intrinsic correlations with cortical regions (see Figure S6 for the corresponding distance matrix).

deviant stimuli may occur for a particular level of tonic LC activity, when the subject is sufficiently focused (and not too much) on the current task.[69,70] In this study, we reported baseline-corrected analyses (epoch-based analyses) to focus on the phasic component and suppress the additive effect of tonic fluctuations (but ignoring potential non-linear effects previously reported in spiking activity of LC neurons[69] and in pupil size[71]). In contrast, the non-baseline-corrected analyses (see FIR and GLM analyses in supplemental information) also focus on the phasic component but ignore the tonic fluctuations (the ones above 1/128 Hz that remain after preprocessing). We note that our results are consistent across baseline and non-baseline-corrected analyses, which suggests in retrospect that the detection of rare patterns that manifests itself in an increased arousal of various brain structures was not masked by fast (above 1/128 Hz) fluctuations of tonic arousal levels (which would have penalized the non-baseline-corrected analyses).

The current work will, in addition, be of methodological interest to researchers interested in the measure of LC activity with fMRI and more indirectly with pupillometry. The possibility to estimate the LC activity with fMRI is a contentious issue; doing so requires dedicated methods such as subject-specific anatomical delineation of the LC (see, e.g., technical comments[45,72]). We report a comparison of results obtained with a subject-specific delineation and a probabilistic, normalized anatomical atlas of the LC. Although both analyses showed an effect of global deviant patterns, this effect interacted with the local pattern type and was actually driven by the rare xxxxY pattern when using the atlas (there is no such interaction when using subject-specific delineation or pupil size). Given what is observed in other brain regions and the pupil, and previous work on the LC, we assume that the LC responds to both types of global deviants and thus that the results obtained with subject-specific delineation are closer to the ground truth. In other words, using a subject-specific delineation (rather than an atlas) seems necessary in fMRI studies of the LC, despite being time and resource consuming. We also propose that the effect of the rare xxxxx patterns could be a quality check of correct identification of the LC region, possibly in a trimmed-down version of the local-global paradigm that only presents rare xxxxx among frequent xxxxY. A response to rare xxxxx patterns seems a more stringent test than a response to rare xxxxY patterns (as in oddball tasks) because we found responses to rare xxxxY patterns, but not rare xxxxx patterns, non-specifically across the pons (see Figure S4).

Our results are also informative concerning the use of peripheral arousal (measured as non-luminance-based change in pupil size) as an approximation of central arousal (more precisely, LC activity). The sensitivity of pupil size to LC activity is demonstrated on the basis of direct LC recording in non-human animals.[39,73] However, those studies also demonstrated that this correlation is not specific to LC activity but is also related to central acetylcholine[39] and serotonin[40] levels. A consequence of this lack of specificity is that changes in pupil dilation may not reflect changes in LC activity.[36] Here we found an effect of the global deviance, without interaction with the local deviance, in both pupil size and fMRI activity in the LC region, suggesting that peripheral and central arousals are similar. Note that those similar effects could arise from the LC influencing pupil size (e.g., via the intermediolateral cell column, the Edinger-Westphal nucleus, or the SC notably[37]) or from a common input (e.g., the nucleus gigantocellularis that activates both the LC and the autonomic system[4]). Those two hypotheses would have been supported by correlated responses to global deviance in the pupil and the LC region, but we did not find such a relationship, which was significant only between pupil size and fMRI activity in the SN/VTA. This null result in the LC is not evidence for the absence of a relationship, notably because our analyses were limited by the small number of included trials and participants. The result found for the SN/VTA (which replicates the one from de Gee et al.[38] in a different task) could simply be due to better data quality, this region being much larger than the LC (here, all effects were stronger in the SN/VTA than in the LC). The SN/VTA has no direct connection to the systems controlling pupil size,[37] but it receives direct input from the LC[4]; the effect found in the SN/VTA could thus be due to an effect in the LC that our data failed to detect.

Overall, the current study showed deviant-related responses to an effect of deviance that generalize across two types (stimulus probability and sequence structure) in many subcortical regions, including neuromodulatory centers, and several cortical regions. Our results are consistent with the idea that the detection of task-relevant deviant sound patterns triggers the arousal

system through the activity of the LC. The LC likely gets inputs signaling the occurrence of a task-relevant event from higher regions (e.g., frontal areas) and in turn broadcasts surprise signals across the entire brain. Future work with better temporal resolution will need to determine the direction of neural signals between the interconnected neuromodulatory centers, other subcortical structures, and cortical areas that subtend a hierarchy of deviance mechanisms.

Limitations of the study

The present study has several limitations. Because of the task structure, the number of global deviant patterns is necessarily small (88 patterns per type of global deviant per participant), therefore analyses comparing signals between the two types of deviant may suffer from a lack of statistical power. The number of voxels corresponding to the anatomically delineated LC is also small, and furthermore different across participants (from 4 to 9 voxels in fMRI data), potentially contributing substantial between-subject variability. The detection of global deviants is confounded by their task relevance, and future studies should disentangle these two aspects. Finally, we did not perform sex- and gender-based analyses.

STAR★METHODS

Detailed methods are provided in the online version of this paper and include the following:

- KEY RESOURCES TABLE
- RESOURCE AVAILABILITY
  - Lead contact
  - Materials availability
  - Data and code availability
- EXPERIMENTAL MODEL AND STUDY PARTICIPANT DETAILS
- METHOD DETAILS
  - Stimuli and task
  - MRI data collection and preprocessing
  - Physiological data collection and preprocessing
  - Pupil size data collection and preprocessing
- QUANTIFICATION AND STATISTICAL ANALYSIS
  - Definition of regions of interest (ROIs) and preprocessing
  - Epoch-based analyses of fMRI signals, baseline correction
  - Connectivity analysis and clustering
  - Finite Impulse Response (FIR) analyses
  - Generalized linear model (GLM) analyses
  - Correction for multiple comparisons across time

SUPPLEMENTAL INFORMATION

Supplemental information can be found online at https://doi.org/10.1016/j.celrep.2023.113405.

ACKNOWLEDGMENTS

This work was funded by the ANR #18-CE37-0010-01 CONFI-LEARN and by the ERC StG NEURAL-PROB #947105 to F. Meyniel. The MRI facility was also partly supported by the European Union (FEDER-2007-2013, agreement #91-2015-004).

AUTHOR CONTRIBUTIONS

F. Meyniel, A.M., A.A., and F. Mauconduit designed the MRI sequences. A.M. collected and analyzed the data. A.M., J.W.d.G., T.H.D., and F. Meyniel wrote the manuscript.

DECLARATION OF INTERESTS

The authors declare no competing interests.

Received: July 21, 2022
Revised: August 10, 2023
Accepted: October 24, 2023
Published: November 10, 2023

REFERENCES

1. Soltani, A., and Izquierdo, A. (2019). Adaptive learning under expected and unexpected uncertainty. Nat. Rev. Neurosci. *20*, 635–644.
2. Devauges, V., and Sara, S.J. (1990). Activation of the noradrenergic system facilitates an attentional shift in the rat. Behav. Brain Res. *39*, 19–28.
3. Janitzky, K., Lippert, M.T., Engelhorn, A., Tegtmeier, J., Goldschmidt, J., Heinze, H.J., and Ohl, F.W. (2015). Optogenetic silencing of locus coeruleus activity in mice impairs cognitive flexibility in an attentional set-shifting task. Front. Behav. Neurosci. *9*, 286.
4. Sara, S.J., and Bouret, S. (2012). Orienting and reorienting: the locus coeruleus mediates cognition through arousal. Neuron *76*, 130–141.
5. Aston-Jones, G., Rajkowski, J., Kubiak, P., and Alexinsky, T. (1994). Locus coeruleus neurons in monkey are selectively activated by attended cues in a vigilance task. J. Neurosci. *14*, 4467–4480.
6. Hervé-Minvielle, A., and Sara, S.J. (1995). Rapid habituation of auditory responses of locus coeruleus cells in anaesthetized and awake rats. Neuroreport *6*, 1363–1368.
7. Sara, S.J., Vankov, A., and Hervé, A. (1994). Locus coeruleus-evoked responses in behaving rats: a clue to the role of noradrenaline in memory. Brain Res. Bull. *35*, 457–465.
8. Poe, G.R., Foote, S., Eschenko, O., Johansen, J.P., Bouret, S., Aston-Jones, G., Harley, C.W., Manahan-Vaughan, D., Weinshenker, D., Valentino, R., et al. (2020). Locus coeruleus: a new look at the blue spot. Nat. Rev. Neurosci. *21*, 644–659.
9. Rajkowski, J., Kubiak, P., and Aston-Jones, G. (1994). Locus coeruleus activity in monkey: phasic and tonic changes are associated with altered vigilance. Brain Res. Bull. *35*, 607–616.
10. Aston-Jones, G., Rajkowski, J., and Kubiak, P. (1997). Conditioned responses of monkey locus coeruleus neurons anticipate acquisition of discriminative behavior in a vigilance task. Neuroscience *80*, 697–715.
11. Foote, S.L., Aston-Jones, G., and Bloom, F.E. (1980). Impulse activity of locus coeruleus neurons in awake rats and monkeys is a function of sensory stimulation and arousal. Proc. Natl. Acad. Sci. USA *77*, 3033–3037.
12. Swick, D., Pineda, J.A., Schacher, S., and Foote, S.L. (1994). Locus coeruleus neuronal activity in awake monkeys: relationship to auditory P300-like potentials and spontaneous EEG. Exp. Brain Res. *101*, 86–92.
13. Krebs, R.M., Park, H.R.P., Bombeke, K., and Boehler, C.N. (2018). Modulation of locus coeruleus activity by novel oddball stimuli. Brain Imaging Behav. *12*, 577–584.
14. Murphy, P.R., O'Connell, R.G., O'Sullivan, M., Robertson, I.H., and Balsters, J.H. (2014). Pupil diameter covaries with BOLD activity in human locus coeruleus. Hum. Brain Mapp. *35*, 4140–4154.
15. Debener, S., Makeig, S., Delorme, A., and Engel, A.K. (2005). What is novel in the novelty oddball paradigm? Functional significance of the novelty P3

event-related potential as revealed by independent component analysis. Brain Res. Cogn. Brain Res. 22, 309–321.

16. Schomaker, J., and Meeter, M. (2015). Short- and long-lasting consequences of novelty, deviance and surprise on brain and cognition. Neurosci. Biobehav. Rev. 55, 268–279.
17. Reichardt, R., Polner, B., and Simor, P. (2020). Novelty Manipulations, Memory Performance, and Predictive Coding: the Role of Unexpectedness. Front. Hum. Neurosci. 14, 152.
18. Verleger, R., and Śmigasiewicz, K. (2016). Do Rare Stimuli Evoke Large P3s by Being Unexpected? A Comparison of Oddball Effects Between Standard-Oddball and Prediction-Oddball Tasks. Adv. Cogn. Psychol. 12, 88–104.
19. Harsay, H.A., Spaan, M., Wijnen, J.G., and Ridderinkhof, K.R. (2012). Error awareness and salience processing in the oddball task: shared neural mechanisms. Front. Hum. Neurosci. 6, 246.
20. Bassareo, V., De Luca, M.A., and Di Chiara, G. (2002). Differential Expression of Motivational Stimulus Properties by Dopamine in Nucleus Accumbens Shell versus Core and Prefrontal Cortex. J. Neurosci. 22, 4709–4719.
21. Brown, S.B.R.E., van der Wee, N.J.A., van Noorden, M.S., Giltay, E.J., and Nieuwenhuis, S. (2015). Noradrenergic and cholinergic modulation of late ERP responses to deviant stimuli. Psychophysiology 52, 1620–1631.
22. Bunzeck, N., and Düzel, E. (2006). Absolute coding of stimulus novelty in the human substantia nigra/VTA. Neuron 51, 369–379.
23. Caldenhove, S., Borghans, L.G.J.M., Blokland, A., and Sambeth, A. (2017). Role of acetylcholine and serotonin in novelty processing using an oddball paradigm. Behav. Brain Res. 331, 199–204.
24. Glimcher, P.W. (2011). Understanding dopamine and reinforcement learning: the dopamine reward prediction error hypothesis. Proc. Natl. Acad. Sci. USA 108, 15647–15654.
25. Pessiglione, M., Seymour, B., Flandin, G., Dolan, R.J., and Frith, C.D. (2006). Dopamine-dependent prediction errors underpin reward-seeking behaviour in humans. Nature 442, 1042–1045.
26. Ungless, M.A. (2004). Dopamine: the salient issue. Trends Neurosci. 27, 702–706.
27. Vazey, E.M., Moorman, D.E., and Aston-Jones, G. (2018). Phasic locus coeruleus activity regulates cortical encoding of salience information. Proc. Natl. Acad. Sci. USA 115, E9439–E9448.
28. Kutlu, M.G., Zachry, J.E., Melugin, P.R., Cajigas, S.A., Chevee, M.F., Kelly, S.J., Kutlu, B., Tian, L., Siciliano, C.A., and Calipari, E.S. (2021). Dopamine release in the nucleus accumbens core signals perceived saliency. Curr. Biol. 31, 4748–4761.e8.
29. Strange, B.A., and Dolan, R.J. (2007). β-adrenergic modulation of oddball responses in humans. Behav. Brain Funct. 3, 29.
30. Nieuwenhuis, S., Aston-Jones, G., and Cohen, J.D. (2005). Decision making, the P3, and the locus coeruleus–norepinephrine system. Psychol. Bull. 131, 510–532.
31. Ahnaou, A., Biermans, R., and Drinkenburg, W.H.I.M. (2018). Cholinergic mechanisms of target oddball stimuli detection: The late ‘P300-like’ event-related potential in rats. Neural Plast. 2018, 4270263.
32. Gilzenrat, M.S., Nieuwenhuis, S., Jepma, M., and Cohen, J.D. (2010). Pupil diameter tracks changes in control state predicted by the adaptive gain theory of locus coeruleus function. Cogn. Affect. Behav. Neurosci. 10, 252–269.
33. Murphy, P.R., Robertson, I.H., Balsters, J.H., and O’connell, R.G. (2011). Pupillometry and P3 index the locus coeruleus-noradrenergic arousal function in humans. Psychophysiology 48, 1532–1543.
34. Nieuwenhuis, S., De Geus, E.J., and Aston-Jones, G. (2011). The anatomical and functional relationship between the P3 and autonomic components of the orienting response. Psychophysiology 48, 162–175.
35. Preuschoff, K., ’t Hart, B.M., and Einhäuser, W. (2011). Pupil Dilation Signals Surprise: Evidence for Noradrenaline’s Role in Decision Making. Front. Neurosci. 5, 115.
36. Megemont, M., McBurney-Lin, J., and Yang, H. (2022). Pupil diameter is not an accurate real-time readout of locus coeruleus activity. Elife 11, e70510.
37. Joshi, S., and Gold, J.I. (2020). Pupil Size as a Window on Neural Substrates of Cognition. Trends Cogn. Sci. 24, 466–480.
38. de Gee, J.W., Colizoli, O., Kloosterman, N.A., Knapen, T., Nieuwenhuis, S., and Donner, T.H. (2017). Dynamic modulation of decision biases by brainstem arousal systems. Elife 6, e23232.
39. Reimer, J., McGinley, M.J., Liu, Y., Rodenkirch, C., Wang, Q., McCormick, D.A., and Tolias, A.S. (2016). Pupil fluctuations track rapid changes in adrenergic and cholinergic activity in cortex. Nat. Commun. 7, 13289.
40. Cazettes, F., Reato, D., Morais, J.P., Renart, A., and Mainen, Z.F. (2021). Phasic Activation of Dorsal Raphe Serotonergic Neurons Increases Pupil Size. Curr. Biol. 31, 192–197.e4.
41. Varazzani, C., San-Galli, A., Gilardeau, S., and Bouret, S. (2015). Noradrenaline and dopamine neurons in the reward/effort trade-off: a direct electrophysiological comparison in behaving monkeys. J. Neurosci. 35, 7866–7877.
42. Liu, K.Y., Marijatta, F., Hämmerer, D., Acosta-Cabronero, J., Düzel, E., and Howard, R.J. (2017). Magnetic resonance imaging of the human locus coeruleus: A systematic review. Neurosci. Biobehav. Rev. 83, 325–355.
43. Ayala, Y.A., and Malmierca, M.S. (2012). Stimulus-specific adaptation and deviance detection in the inferior colliculus. Front. Neural Circuits 6, 89.
44. Malmierca, M.S., Cristaudo, S., Pérez-González, D., and Covey, E. (2009). Stimulus-specific adaptation in the inferior colliculus of the anesthetized rat. J. Neurosci. 29, 5483–5493.
45. Astafiev, S.V., Snyder, A.Z., Shulman, G.L., and Corbetta, M. (2010). Comment on ‘Modafinil Shifts Human Locus Coeruleus to Low-Tonic, High-Phasic Activity During Functional MRI’ and ‘Homeostatic Sleep Pressure and Responses to Sustained Attention in the Suprachiasmatic Area. Science 328, 309.
46. Bekinschtein, T.A., Dehaene, S., Rohaut, B., Tadel, F., Cohen, L., and Naccache, L. (2009). Neural signature of the conscious processing of auditory regularities. Proc. Natl. Acad. Sci. USA 106, 1672–1677.
47. Dehaene, S., Meyniel, F., Wacongne, C., Wang, L., and Pallier, C. (2015). The Neural Representation of Sequences: From Transition Probabilities to Algebraic Patterns and Linguistic Trees. Neuron 88, 2–19.
48. Heilbron, M., and Chait, M. (2018). Great Expectations: Is there Evidence for Predictive Coding in Auditory Cortex? Neuroscience 389, 54–73.
49. King, J.R., Faugeras, F., Gramfort, A., Schurger, A., El Karoui, I., Sitt, J.D., Rohaut, B., Wacongne, C., Labyt, E., Bekinschtein, T., et al. (2013). Single-trial decoding of auditory novelty responses facilitates the detection of residual consciousness. Neuroimage 83, 726–738.
50. Strauss, M., Sitt, J.D., King, J.R., Elbaz, M., Azizi, L., Buiatti, M., Naccache, L., van Wassenhove, V., and Dehaene, S. (2015). Disruption of hierarchical predictive coding during sleep. Proc. Natl. Acad. Sci. USA 112, E1353–E1362.
51. Quirins, M., Marois, C., Valente, M., Seassau, M., Weiss, N., El Karoui, I., Hochmann, J.R., and Naccache, L. (2018). Conscious processing of auditory regularities induces a pupil dilation. Sci. Rep. 8, 14819.
52. Friston, K.J. (2003). Statistical Parametric Mapping. In Neuroscience Databases, R. Kötter, ed. (Springer), pp. 237–250. https://doi.org/10.1007/978-1-4615-1079-6_16.
53. Destrieux, C., Fischl, B., Dale, A., and Halgren, E. (2010). Automatic parcellation of human cortical gyri and sulci using standard anatomical nomenclature. Neuroimage 53, 1–15.
54. Keren, N.I., Taheri, S., Vazey, E.M., Morgan, P.S., Granholm, A.C.E., Aston-Jones, G.S., and Eckert, M.A. (2015). Histologic validation of locus coeruleus MRI contrast in post-mortem tissue. Neuroimage 113, 235–245.
55. Keren, N.I., Lozar, C.T., Harris, K.C., Morgan, P.S., and Eckert, M.A. (2009). In vivo mapping of the human locus coeruleus. Neuroimage 47, 1261–1267.

CellPress
OPEN ACCESS

56. Karoui, I.E., King, J.-R., Sitt, J., Meyniel, F., Van Gaal, S., Hasboun, D., Adam, C., Navarro, V., Baulac, M., Dehaene, S., et al. (2014). Event-Related Potential, Time-frequency, and Functional Connectivity Facets of Local and Global Auditory Novelty Processing: An Intracranial Study in Humans. Cereb. Cortex, bhu143.
57. Wacongne, C., Labyt, E., van Wassenhove, V., Bekinschtein, T., Naccache, L., and Dehaene, S. (2011). Evidence for a hierarchy of predictions and prediction errors in human cortex. Proc. Natl. Acad. Sci. USA *108*, 20754–20759.
58. Dürschmid, S., Edwards, E., Reichert, C., Dewar, C., Hinrichs, H., Heinze, H.J., Kirsch, H.E., Dalal, S.S., Deouell, L.Y., and Knight, R.T. (2016). Hierarchy of prediction errors for auditory events in human temporal and frontal cortex. Proc. Natl. Acad. Sci. USA *113*, 6755–6760.
59. Chao, Z.C., Takaura, K., Wang, L., Fujii, N., and Dehaene, S. (2018). Large-Scale Cortical Networks for Hierarchical Prediction and Prediction Error in the Primate Brain. Neuron *100*, 1252–1266.e3.
60. Siegel, M., Donner, T.H., and Engel, A.K. (2012). Spectral fingerprints of large-scale neuronal interactions. Nat. Rev. Neurosci. *13*, 121–134.
61. Wang, X.-J. (2010). Neurophysiological and computational principles of cortical rhythms in cognition. Physiol. Rev. *90*, 1195–1268.
62. Bastos, A.M., Vezoli, J., Bosman, C.A., Schoffelen, J.M., Oostenveld, R., Dowdall, J.R., De Weerd, P., Kennedy, H., and Fries, P. (2015). Visual Areas Exert Feedforward and Feedback Influences through Distinct Frequency Channels. Neuron *85*, 390–401.
63. Arnsten, A.F., and Goldman-Rakic, P.S. (1984). Selective prefrontal cortical projections to the region of the locus coeruleus and raphe nuclei in the rhesus monkey. Brain Res. *306*, 9–18.
64. Luppi, P.H., Aston-Jones, G., Akaoka, H., Chouvet, G., and Jouvet, M. (1995). Afferent projections to the rat locus coeruleus demonstrated by retrograde and anterograde tracing with cholera-toxin B subunit and Phaseolus vulgaris leucoagglutinin. Neuroscience *65*, 119–160.
65. Faugeras, F., Rohaut, B., Weiss, N., Bekinschtein, T., Galanaud, D., Puybasset, L., Bolgert, F., Sergent, C., Cohen, L., Dehaene, S., and Naccache, L. (2012). Event related potentials elicited by violations of auditory regularities in patients with impaired consciousness. Neuropsychologia *50*, 403–418.
66. Bellet, M.E., Gay, M., Bellet, J., Jarraya, B., Dehaene, S., van Kerkoerle, T., and Panagiotaropoulos, T.I. (2021). Prefrontal neural ensembles encode an internal model of visual sequences and their violations. Preprint at. bioRxiv. https://doi.org/10.1101/2021.10.04.463064.
67. Uhrig, L., Dehaene, S., and Jarraya, B. (2014). A Hierarchy of Responses to Auditory Regularities in the Macaque Brain. J. Neurosci. *34*, 1127–1132.
68. Jiang, Y., Komatsu, M., Chen, Y., Xie, R., Zhang, K., Xia, Y., Gui, P., Liang, Z., and Wang, L. (2022). Constructing the hierarchy of predictive auditory sequences in the marmoset brain. Elife *11*, e74653.
69. Aston-Jones, G., and Cohen, J.D. (2005). An integratice theory of locus coeruleus-norepinephrine function: Adaptive gain and optimal performance. Annu. Rev. Neurosci. *28*, 403–450.
70. Dubois, M., Habicht, J., Michely, J., Moran, R., Dolan, R.J., and Hauser, T.U. (2021). Human complex exploration strategies are enriched by noradrenaline-modulated heuristics. Elife *10*, e59907.
71. Knapen, T., de Gee, J.W., Brascamp, J., Nuiten, S., Hoppenbrouwers, S., and Theeuwes, J. (2016). Cognitive and Ocular Factors Jointly Determine Pupil Responses under Equiluminance. PLoS One *11*, e0155574.
72. Eckert, M.A., Keren, N.I., and Aston-Jones, G. (2010). Looking Forward with the Locus Coeruleus (Science E-Letter).
73. Costa, V.D., and Rudebeck, P.H. (2016). More than Meets the Eye: the Relationship between Pupil Size and Locus Coeruleus Activity. Neuron *89*, 8–10.
74. Brainard, D.H. (1997). The Psychophysics Toolbox. Spat. Vis. *10*, 433–436.
75. Chen, X., Huddleston, D.E., Langley, J., Ahn, S., Barnum, C.J., Factor, S.A., Levey, A.I., and Hu, X. (2014). Simultaneous imaging of locus coeruleus and substantia nigra with a quantitative neuromelanin MRI approach. Magn. Reson. Imaging *32*, 1301–1306.
76. Brooks, J.C.W., Faull, O.K., Pattinson, K.T.S., and Jenkinson, M. (2013). Physiological noise in brainstem FMRI. Front. Hum. Neurosci. *7*, 623.
77. Zaborszky, L., Hoemke, L., Mohlberg, H., Schleicher, A., Amunts, K., and Zilles, K. (2008). Stereotaxic probabilistic maps of the magnocellular cell groups in human basal forebrain. Neuroimage *42*, 1127–1141.
78. Edlow, B.L., Takahashi, E., Wu, O., Benner, T., Dai, G., Bu, L., Grant, P.E., Greer, D.M., Greenberg, S.M., Kinney, H.C., and Folkerth, R.D. (2012). Neuroanatomic connectivity of the human ascending arousal system critical to consciousness and its disorders. J. Neuropathol. Exp. Neurol. *71*, 531–546.
79. Pedregosa, F., Varoquaux, G., Gramfort, A., Michel, V., Thirion, B., Grisel, O., Blondel, M., Prettenhofer, P., Weiss, R., Dubourg, V., and Vanderplas, J. (2012). Scikit-learn: Machine Learning in Python. Preprint at. arXiv, 2825–2830.
80. Gramfort, A., Luessi, M., Larson, E., Engemann, D.A., Strohmeier, D., Brodbeck, C., Goj, R., Jas, M., Brooks, T., Parkkonen, L., and Hämäläinen, M. (2013). MEG and EEG data analysis with MNE-Python. Front. Neurosci. *7*, 267.

STAR★METHODS

KEY RESOURCES TABLE

REAGENT or RESOURCE	SOURCE	IDENTIFIER
Deposited data		
Behavioral, physiological, and pupil data	This article	https://osf.io/td5kp/
MRI data	This article	https://openneuro.org/datasets/ds004808
Software and algorithms		
Python version 3.7	Python Software Foundation	www.python.org
Psychtoolbox 3.0.19.2	Open source	http://psychtoolbox.org/
SPM12	MATLAB (Mathworks)	https://www.fil.ion.ucl.ac.uk/spm/software/spm12/
FSL	Analysis Group, FMRIB, Oxford, UK	https://fsl.fmrib.ox.ac.uk/fsl/fslwiki
Nipype	Open source	https://doi.org/10.5281/zenodo.596855
Octave 6.2.0	Open source	https://octave.org/
sklearn 1.0.2	Open source	https://scikit-learn.org/stable/
MNE Python	Open source	https://mne.tools/stable/index.html
Nilearn 0.9.2	Open source	https://nilearn.github.io/
Original code	This article	https://zenodo.org/record/8414347

RESOURCE AVAILABILITY

Lead contact

Further information and requests for resources and reagents should be directed to and will be fulfilled by the lead contact, Florent Meyniel (florent.meyniel@cea.fr).

Materials availability

This study did not generate new unique reagents.

Data and code availability

- Behavioral, physiological, pupil data are available on https://osf.io/td5kp/. MRI data are available on https://openneuro.org/datasets/ds004808.
- Original code is available on https://zenodo.org/record/8414347.
- Any additional information required to reanalyze the data reported in this work paper is available from the lead contact upon request.

EXPERIMENTAL MODEL AND STUDY PARTICIPANT DETAILS

Twenty four participants (10 women as per sex assigned at birth; gender was not collected) recruited in the general population and aged between 20 and 36 years (mean = 27.04, SD = 4.69) were enrolled in the experiment. Ancestry, race and ethnicity were not collected. This protocol was approved by a national ethics committee (Comité de Protection des Personnes Ile de France 3, approval #2018-A03195-50). Participants gave their written informed consent prior to participating in the study. Participants receive monetary compensation for their participation (80€ for 2 h). They were right-handed based on self-report and had normal or corrected-to-normal vision.

METHOD DETAILS

Stimuli and task

The task included 4 sessions of 10 min each and was run using Octave (version 6.1.0) and the Psychtoolbox[74] in the scanner. It was the same task as used in.[46] Stimuli are short auditory tones composed by 3 sinusoidal tones resulting in either a low-pitched sound (stimulus A composed by 350, 700, and 1400 Hz sinusoides) or a high-pitched sound (stimulus B: 500 Hz, 1000 Hz, and 2000 Hz

sinusoides). Stimuli were presented in a sequence of patterns separated by pauses. A pattern consisted in four identical tones and a fifth that could be either the same (xxxxx; within-pattern standard, i.e., *local standard*) or different (xxxxY; within-pattern deviant, i.e., *local deviant*). The assignment of tones and patterns were counterbalanced across blocks (block of AAAAA and AAAAB vs. BBBBB and BBBBA). The duration of each tone was 50 ms and pattern duration was 650 ms with an inter-pattern interval of 1.500ms. During the habituation phase, participants were first exposed to only one pattern. During the test phase, participants were presented with either the same pattern as during habituation in 80% of the cases (*frequent pattern*) or with the other pattern in 20% of the cases (*rare pattern*). Figure 1A depicts a schematic representation of the task.

Each session included 2 blocks in counterbalanced order: one where the habituation pattern was a local standard pattern (denoted xxxxx block) and one where it was the local deviant pattern (denoted xxxxY block). One block included 135 patterns (22 rare patterns and 113 frequent patterns including 25 ones for the habituation phase). During the task, participants had to listen to the pattern and count the number of rare patterns.

MRI data collection and preprocessing

MRI data were acquired on a 3 Tesla scanner (Siemens, Prisma) with a 64-channel coil. In order to maximize the signal-to-noise ratio in LC, we acquired partial-brain functional echo planar images (EPI) images centered on the brainstem and oriented perpendicular to the floor of the fourth ventricle (and thus, main axis of the LC). We used the following parameters: TR = 1.25 s, TE = 30 ms, flip angle = 65°, 28 interleaved slices with a slice thickness of 3 mm and a multiband factor of 2. In-plane resolution was 2.0 × 2.0 mm. The encoding phase direction was from anterior to posterior. To estimate distortions, we acquired two volumes with opposite phase encoding directions. One volume was in the anterior to posterior direction (AP) and the other was in the other direction (PA), with TR = 4,800 ms, TE = 54 ms.

Two partial-brain Turbo Spin Echo (TSE) structural images, sometimes referred to as neuromelanin-sensitive[75] were acquired: one centered on the LC[38] and others centered on the SN/VTA. Images were acquired with an in-plane resolution of 0.7 × 0.7 mm and reconstructed at 0.35x0.35 (TR = 675 ms, TE = 12 ms). We acquired 14 slices per TSE, slice thickness was 2 mm, oriented perpendicular to the floor of the fourth ventricle. We also acquired a whole-brain structural T1 image with an MPRAGE sequence for anatomical co-registration and the delineation of the IC and the SC with in-plane resolution of 1 × 1 mm and a slice thickness of 1 mm (TR = 2,300 ms, TE = 2.98 ms).

All preprocessing steps relied on SPM12 (Wellcome Trust Center for Neuroimaging, University College London) except the TOPUP correction that relied on FSL, using the python/FSL and python/SPM interfaces afforded by Nipype (https://doi.org/10.5281/zenodo.596855). Slice-timing correction was referenced to the middle of each TR. Volumes were realigned onto the first volume of each session, and then onto the first volume of the first session. We also performed a TOPUP correction that estimates the susceptibility field using the AP/PA volumes and unwraps EPI images. Different coregistrations were made for different types of analyses. For those in native space analyses, EPI images were coregistered with the TSE images (either with the one centered on the LC to extract LC data, or the one centered on the SN/VTA to extract SN/VTA data) or with the T1 image (to extract IC and SC data). For normalized space analyses, the T1 image was first coregistered to the TSE image before normalization performed using the standard SPM template in the Montreal Neurological Institute (MNI) space.

Physiological data collection and preprocessing

During the task, we recorded cardiac rhythm with a pulse oximeter and respiration with a belt. We modeled physiological signals using FSL PNM[76] that creates physiological regressors for each slice of each volume. We selected estimates for the reference slice used in the slice-timing correction. We defined orders for each component as follows: 4 for the cardiac component, 3 for the respiratory component, and 1 for the interaction between the two. The total number of regressors modeling physiological signals was 18. One participant had no physiological recordings due to technical issues.

Pupil size data collection and preprocessing

Pupil size was also recorded during scanning using an MRI-compatible EyeLink 1000 system. On raw data we performed the following preprocessing steps: (1) add a margin of 50 ms before and after the blinks detected by the EyeLink system, (2) interpolate the signal linearly within each blink, (3) low-pass filter (5 Hz) the data, (4) epoch the data within −0.5 to 3 s relative to each stimulus onset, (5) exclude epochs with a total blink duration exceeding 20% of the data. It is difficult to measure pupil size in the MRI scanner due to the distance between the eyes and the camera, the use of a mirror, and the partial occlusion by the antenna around the participant's head. We excluded 11 participants for whom pupil size data was available on less than 20% of epochs. The number of participants included in the analyses related to pupil size was therefore 13.

QUANTIFICATION AND STATISTICAL ANALYSIS

Definition of regions of interest (ROIs) and preprocessing

We delineated by hand for each participant ROIs in native space using the TSE images for the LC and SN/VTA (see Figure 1B for an example for one participant see section 8 of Supplementary Results for all participants), and the fourth ventricle, and the T1 image (for the IC and the SC). All masks were resampled to match the EPI resolution resulting in a probabilistic mask that was then transformed

into a binary mask. Threshold probability of being part of the ROI was 0.05. We extracted time series from the EPI images using these masks. Anatomical landmark for the BF, the RN and to a lesser extent the hippocampus are less reliable in TSE and T1 images, thus, we used anatomical atlases in normalized space (maps from[77] for the BF; the Harvard Ascending Arousal Network atlas from[78] for the RN; the Harvard-Oxford cortical and subcortical structural atlases in FSL for the hippocampus). For comparisons between native and normalized space, we also used an anatomical atlas for the LC.[54] Temporal signal-to-noise ratio in our main ROI - the native space LC - was 43.48 (SD = 5.46) (computed with the module TSNR from nipype, using a polynomial detrending of order 3).

For cortical ROIs, we used a complete parcellation of the whole brain[53] into 75 regions. For all these regions, we performed epoch-based analyses (see below) and reported the effect of global deviants in each region (see supplemental information). From these regions, we selected the superior temporal gyrus and the superior temporal sulcus as the auditory processing regions, the calcarine for a primary visual processing region, and the gyrus rectus as a part of the default mode network. For each ROI, we preprocessed the signal by high-pass filtering (1/128 Hz).

Epoch-based analyses of fMRI signals, baseline correction

We performed epoch-based analyses on fMRI time-series extracted from each ROI. We first linearly regressed out potential confounding variables (movement parameters, the time-series extracted from the fourth ventricle, and physiological regressors), and z-scored the residual signal per session. This signal was then upsampled (factor 1000, linear interpolation) and data was then epoched around each stimulus onset (time window: −2 s to 12) for each participant. Then, the baseline signal was subtracted from each epoch using a time window of −2 s–0 s.

Connectivity analysis and clustering

We estimated functional connectivity by calculating subject-level correlations on fMRI time-series extracted from each ROI across regions. We first linearly regressed out potential confounding variables (movement parameters, the time-series extracted from the fourth ventricle, and physiological regressors) as well as the effect of the stimuli, and z-scored the residual signal per session. We performed 2 types of connectivity analyses: one across subcortical regions (LC, SN/VTA, BF, RF, SC, and IC) and one between each of these regions and cortical regions with substantial coverage in our field of view (>50% of the region, see Supplementary results). We then performed hierarchical clustering based on those correlation matrices, using the module AgglomerativeClustering from scikit-learn.[79] The correlation matrix between subcortical structures was used as a distance metric (correlation distance), and the correlation matrix between subcortical and cortical structures was used as a feature matrix on which cosine distance was computed. The clustering algorithm used these distance matrices to cluster subcortical regions, using the average of distances as a criterion to merge clusters.

Finally, we repeated these 2 analyses for blocks with the rare xxxxx and blocks with the rare xxxxY. For each subject, we compared subcortico-subcortical correlations across blocks on the one hand, and cosine distance (between subcortico-cortical correlations) across blocks on the other hand. We performed a t test at the group level to assess significance.

Finite Impulse Response (FIR) analyses

FIR analyses model a number of successive post-stimulus time steps that allow to take into account stimuli that are presented to the participant during the time window of interest, controlling for potential superposition of effects. As for epoch-based analyses, the predefined time-window was from 0 s to 12 s around the onset of patterns and we added additional regressors (movement parameters, the fourth ventricle time-series, and physiological regressors) in our model. For these analyses, the fMRI signal was upsampled with a factor of 5. FIR analyses make no assumptions about the hemodynamic response. We only modeled the effect of rare patterns. At the group level, we tested whether the parameter estimates for these patterns differed from 0 by using a one sample cluster permutation test (cluster-forming and cluster-level alphas of 0.05, two-tailed tests, 10,000 permutations).

Generalized linear model (GLM) analyses

As for FIR analyses, GLM-based analyses control for potential superposition of effects but assume the hemodynamic response to be canonical. One GLM was estimated on time-series per ROIs. The design matrix included the 4 types of patterns convolved with the canonical hemodynamic response function (as modeled in SPM) as well as additional regressors corresponding to movement parameters, time series in the fourth ventricle, and physiological regressors. Parameters (betas) were estimated at the subject level with an auto-regressive AR(1) model. We then computed the difference in parameter estimates between rare and frequent patterns, and tested for its significance (against 0) at the group-level using a t test.

Correction for multiple comparisons across time

For epoch-based analyses (of fMRI signals and pupil size) and FIR analyses, we performed non-parametric cluster-level paired t-tests. As both require multiple comparisons across peri-stimulus times, family-wise error (FWE) correction for multiple comparisons was computed using a cluster-based permutation test (cluster-forming and cluster-level alphas of 0.05, two-tailed tests, 10,000 permutations) with the ‘mne’ package in Python.[80]

Cell Reports, Volume *42*

Supplemental information

Brainstem fMRI signaling of surprise

across different types of deviant stimuli

Audrey Mazancieux, Franck Mauconduit, Alexis Amadon, Jan Willem de Gee, Tobias H. Donner, and Florent Meyniel

Supplemental information

Table S1. Maximum Cohen's d for significant clusters after FWE correction (pFWE < 0.05) and coverage for each parcel (i.e. region) of the Destrieux parcellation. Bold font indicates the regions included in the functional connectivity analysis (i.e., more than 50% coverage). Blue regions are regions included in the manuscript.

Code	Region	Coverage	Maximum Cohen'd
**1**	**Frontomarginal gyrus and sulcus**	**0.99**	**0**
**2**	**Inferior occipital gyrus and sulcus**	**1**	**0**
3	Paracentral lobule and sulcus	0	0
**4**	**Subcentral gyrus and sulcus**	**0.52**	**0.96**
**5**	**Transverse frontopolar gyrus and sulcus**	**0.58**	**0**
6	Anterior cingulate gyrus and sulcus	0.05	0
7	Middle-anterior cingulate gyrus and sulcus	0	0
8	Middle-posterior cingulate gyrus and sulcus	0.01	0
**9**	**Posterior-dorsal part of the cingulate gyrus**	**0.55**	**0**
**10**	**Posterior-ventral part of the cingulate gyrus**	**1**	**0**
**11**	**Cuneus**	**0.65**	**1.21**
12	Opercular part of the inferior frontal gyrus	0.34	1.70
13	Orbital part of the inferior frontal gyrus	0.42	**0.52**
14	Triangular part of the inferior frontal gyrus	0.03	0.42
15	Middle frontal gyrus	0.06	0.57
16	Superior frontal gyrus	0.03	0
**17**	**Long insular gyrus and central sulcus of the insula**	**0.98**	**0.65**
**18**	**Short insular gyrus**	**0.72**	**1.93**
**19**	**Middle occipital gyrus**	**0.75**	**0**
20	Superior occipital gyrus	0.08	0
21	Lateral occipito-temporal gyrus	1	0
**22**	**Lingual gyrus**	**1**	**0.97**
**23**	**Parahippocampal gyrus**	**1**	**0**
**24**	**Orbital gyrus**	**0.67**	**0.46**
**25**	**Angular gyrus**	**0.79**	**0**
**26**	**Supramarginal gyrus**	**0.55**	**1.14**
27	Superior parietal lobule	0.06	1.24
28	Postcentral gyrus	0.001	0
29	Precentral gyrus	0.001	1.17
**30**	**Precuneus**	**0.51**	**0**
**31**	**Rectus gyrus**	**0.72**	**0**
**32**	**Subcallosal gyrus**	**0.98**	**0.70**
**33**	**Anterior transverse temporal gyrus**	**1**	**1.30**
**34**	**Lateral aspect of the superior temporal gyrus**	**1**	**2.04**
**35**	**Planum polare of the superior temporal gyrus**	**1**	**1.21**
**36**	**Superior temporal gyrus**	**0.96**	**1.12**
**37**	**Inferior temporal gyrus**	**1**	**0.97**
**38**	**Middle temporal gyrus**	**1**	**0.45**
39	Horizontal ramus of the anterior segment of the lateral sulcus	0.01	0.90
**40**	**Vertical ramus of the anterior segment of the lateral sulcus**	**1**	**0**
**41**	**Posterior ramus of the lateral sulcus**	**0.95**	**0.84**
42	Occipital pole	0.46	0.96
**43**	**Temporal pole**	**1**	**0**
**44**	**Calcarine sulcus**	**1**	**1.29**

45	Central sulcus	0.006	0.79
46	Marginal branch (or part) of the cingulate sulcus	0.04	0
**47**	**Anterior circular insula sulcus**	**0.65**	**1.79**
**48**	**Inferior circular insula**	**1**	**1.31**
49	Superior segment of the circular sulcus of the insula	0.19	1.36,
**50**	**Anterior transverse collateral sulcus**	**1**	**0**
**51**	**Posterior transverse collateral sulcus**	**1**	**0**
52	Interior frontal sulcus	0.15	0.68
53	Middle frontal sulcus	0.08	0.47
54	Superior frontal sulcus	0	0
**55**	**Sulcus intermedius primus**	**1**	**1.02**
**56**	**Intraparietal sulcus and transverse parietal sulci**	**0.70**	**1.69**
**57**	**Middle occipital sulcus and lunatus sulcus**	**0.91**	**0.49**
**58**	**Superior occipital sulcus and transverse occipital sulcus**	**0.86**	**0.47**
**59**	**Anterior occipital sulcus**	**1**	**0**
**60**	**Lateral occipito-temporal sulcus**	**1**	**0**
**61**	**Medial occipito-temporal sulcus and lingual sulcus**	**1**	**0**
62	Lateral orbital sulcus	0.01	0.61
**63**	**Medial orbital sulcus**	**0.99**	**1.28**
**64**	**Orbital sulci (H-shaped sulci)**	**0.53**	**0.67**
**65**	**Parieto-occipital sulcus**	**0.96**	**0.64**
**66**	**Pericallosal sulcus**	**0.52**	**1.08**
67	Postcentral sulcus	0.05	1.61
68	Interior precentral sulcus	0.005	2.26
69	Superior precentral sulcus	0	0
70	Suborbital sulcus	0.38	0.53
**71**	**Subparietal sulcus**	**0.72**	**0**
**72**	**Interior temporal sulcus**	**1**	**0.91**
**73**	**Superior temporal sulcus**	**1**	**1.48**
**74**	**Transverse temporal sulcus**	**1**	**1.57**

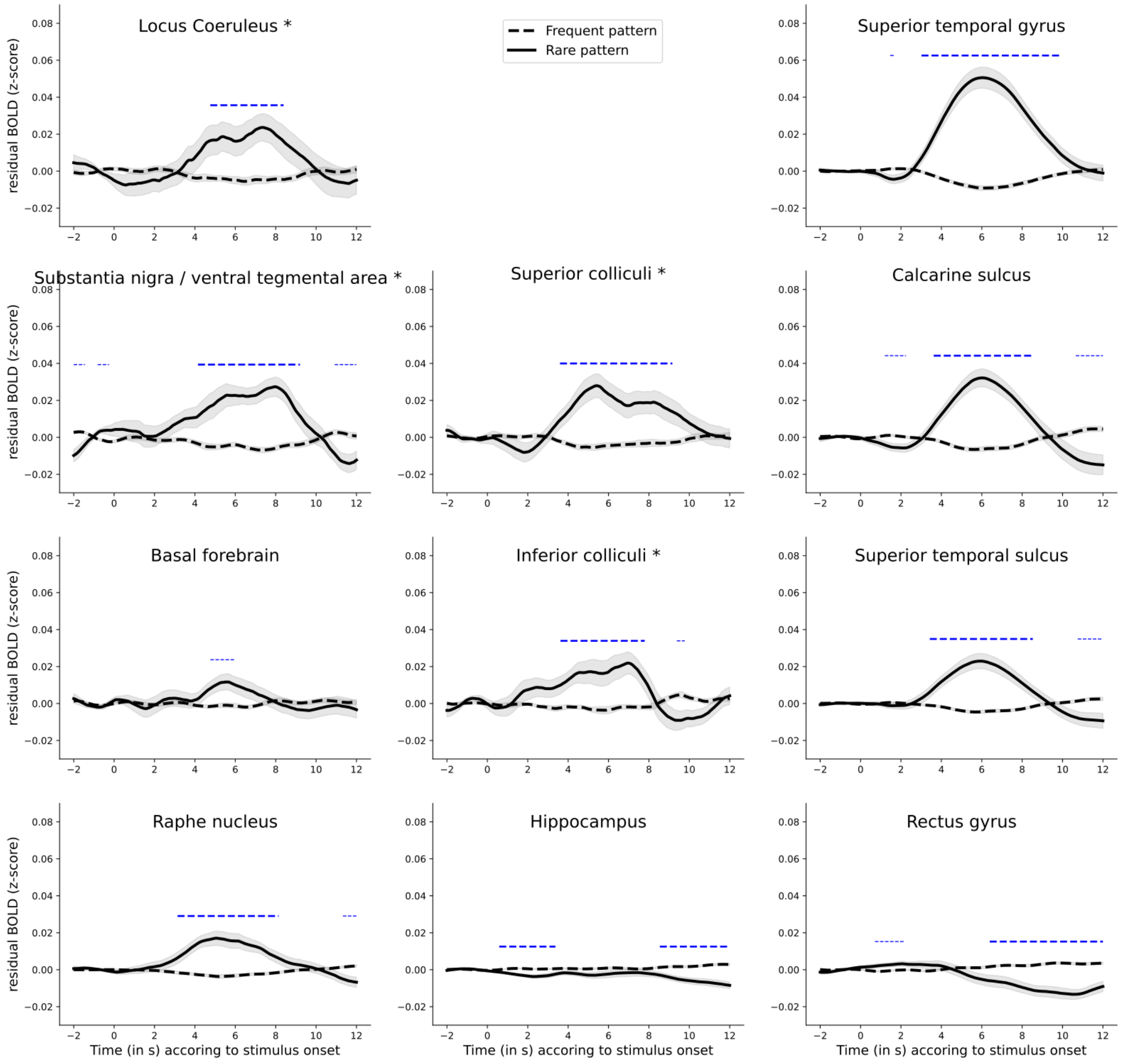

Figure S1. Time course of fMRI activity (z-score) evoked by rare patterns (bold line) and frequent patterns (dashed-line) in different brain structures. The first column shows neuromodulator nuclei, the second column other subcortical ROIs, and the third column cortical ROIs. Horizontal dashed blue lines correspond to clusters of significant difference ($p < 0.05$) and bold dashed blue lines to significant clusters after FWE correction ($p_{FWE} < 0.05$).

Table S2. Statistics for the effect of rare patterns in FIR analyses (max t-values, max p-values, max Cohen's d, and p-values for clusters with FWE correction), for all ROIs. When several clusters have been identified, the one with the higher max Cohen's d is reported. Note that for these analyses, the significant cluster identified for the LC did not reach significance when corrected for multiple comparisons across time.

Neuromodulation-related nuclei	Other subcortical nuclei	Cortical areas
**LC**<br>t_{max} = 4.11<br>p_{max} < 0.001<br>d_{max} = 0.84<br>cluster p_{FWE} < 0.001	**Superior colliculi**<br>t_{max} = 4.19<br>p_{max} < 0.001<br>d_{max} = 0.86<br>cluster p_{FWE} = 0.002	**Superior temporal gyrus**<br>t_{max} = 8.86<br>p_{max} = < 0.001<br>d_{max} = 1.82<br>cluster p_{FWE} = < 0.001
**SN/VTA**<br>t_{max} = 6.28<br>p_{max} < 0.001<br>d_{max} = 1.28<br>cluster p_{FWE} < 0.001	**Inferior colliculi**<br>t_{max} = 4.36<br>p_{max} < 0.001<br>d_{max} = 0.89<br>cluster p_{FWE} < 0.001	**Calcarine sulcus**<br>t_{max} = 5.75<br>p_{max} = < 0.001<br>d_{max} = 1.17<br>cluster p_{FWE} = <0 .001
**BF**<br>t_{max} = 4.72<br>p_{max} < 0.001<br>d_{max} = 0.94<br>cluster p_{FWE} < 0.001	**Hippocampus**<br>t_{max} = 2.27<br>p_{max} = 0.033<br>d_{max} = 0.46<br>cluster p_{FWE} = 0.277	**Superior temporal sulcus**<br>t_{max} = 5.74<br>p_{max} = < 0.001<br>d_{max} = 1.17<br>cluster p_{FWE} = < 0.001
**RN**<br>t_{max} = 5.03<br>p_{max} < 0.001<br>d_{max} = 1.03<br>cluster p_{FWE} < 0.001		**Rectus gyrus**<br>t_{max} = -7.83<br>p_{max} = < 0.001<br>d_{max} = -1.60<br>cluster p_{FWE} = < 0.001

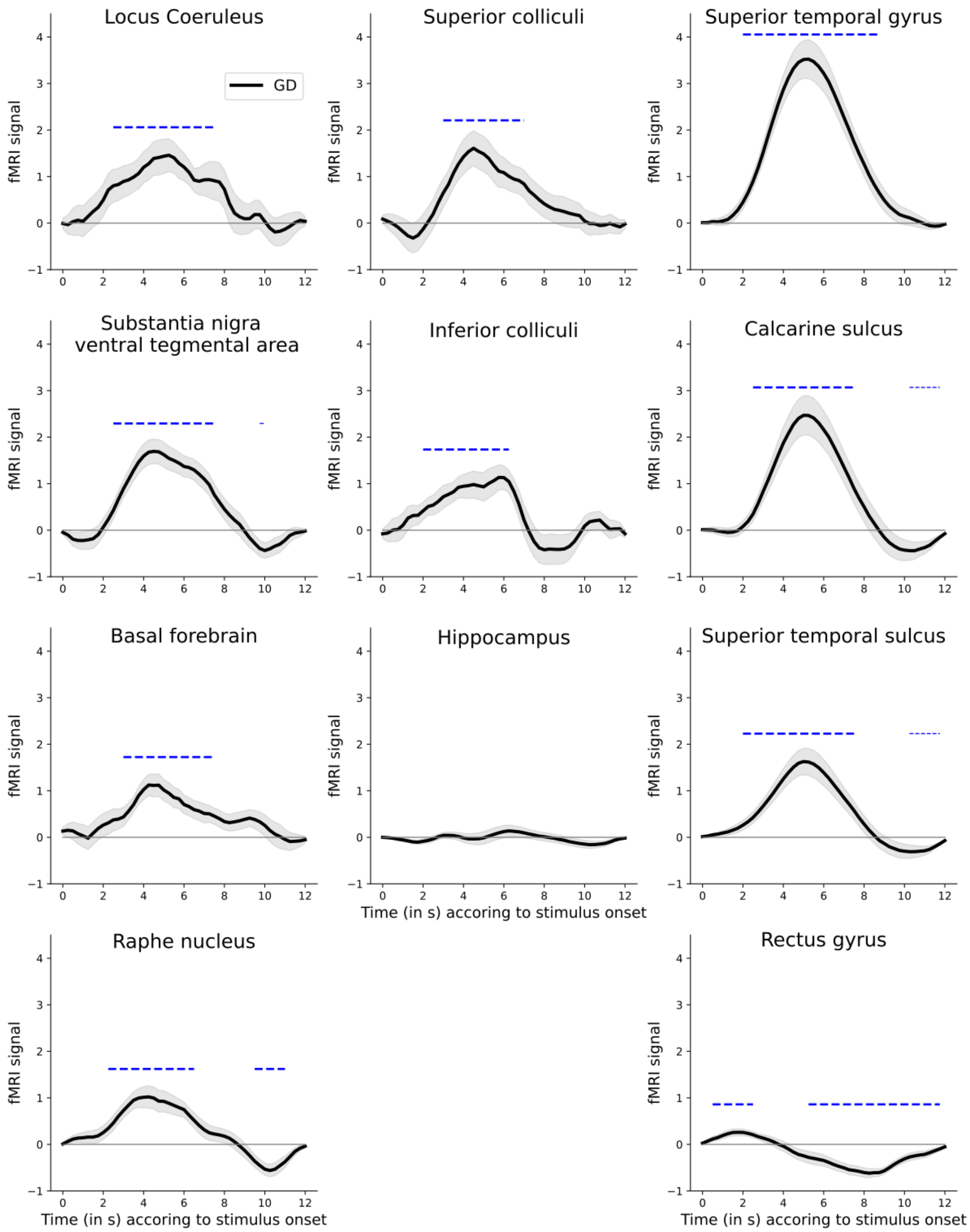

Figure S2. Time course of fMRI activity estimated using a FIR model for rare patterns. The first column shows neuromodulator nuclei, the second column other subcortical ROIs, and the third column cortical ROIs. Error shading is standard error. Blue dashed lines indicate significant clusters different from 0 (p_{FWE}<0.05).

Table S3. Statistics for the difference between the effects of rare and frequent patterns in GLM analyses (t-values, p-values, and Cohen's d), for all ROIs.

**Neuromodulation-related nuclei**	**Other subcortical nuclei**	**Cortical areas**
**LC**<br>$t(23) = 4.59$<br>$p < 0.001$<br>$d = 0.94$	**Superior colliculi**<br>$t(19) = 5.52$<br>$p < 0.001$<br>$d = 1.13$	**Superior temporal gyrus**<br>$t(23) = 9.20$<br>$p < 0.001$<br>$d = 1.88$
**SN/VTA**<br>$t(23) = 5.44$<br>$p < 0.001$<br>$d = 1.11$	**Inferior colliculi**<br>$t(23) = 2.53$<br>$p = 0.019$<br>$d = 0.52$	**Calcarine sulcus**<br>$t(23) = 6.51$<br>$p < 0.001$<br>$d = 1.33$
**BF**<br>$t(23) = 3.98$<br>$p = 0.001$<br>$d = 0.81$	**Hippocampus**<br>$t(23) = 1.50$<br>$p = 0.148$<br>$d = 0.31$	**Superior temporal sulcus**<br>$t(19) = 5.46$<br>$p < 0.001$<br>$d = 1.11$
**RN**<br>$t(19) = 4.41$<br>$p < 0.001$<br>$d = 0.90$		**Rectus gyrus**<br>$t(23) = -3.29$<br>$p = 0.003$<br>$d = -0.67$

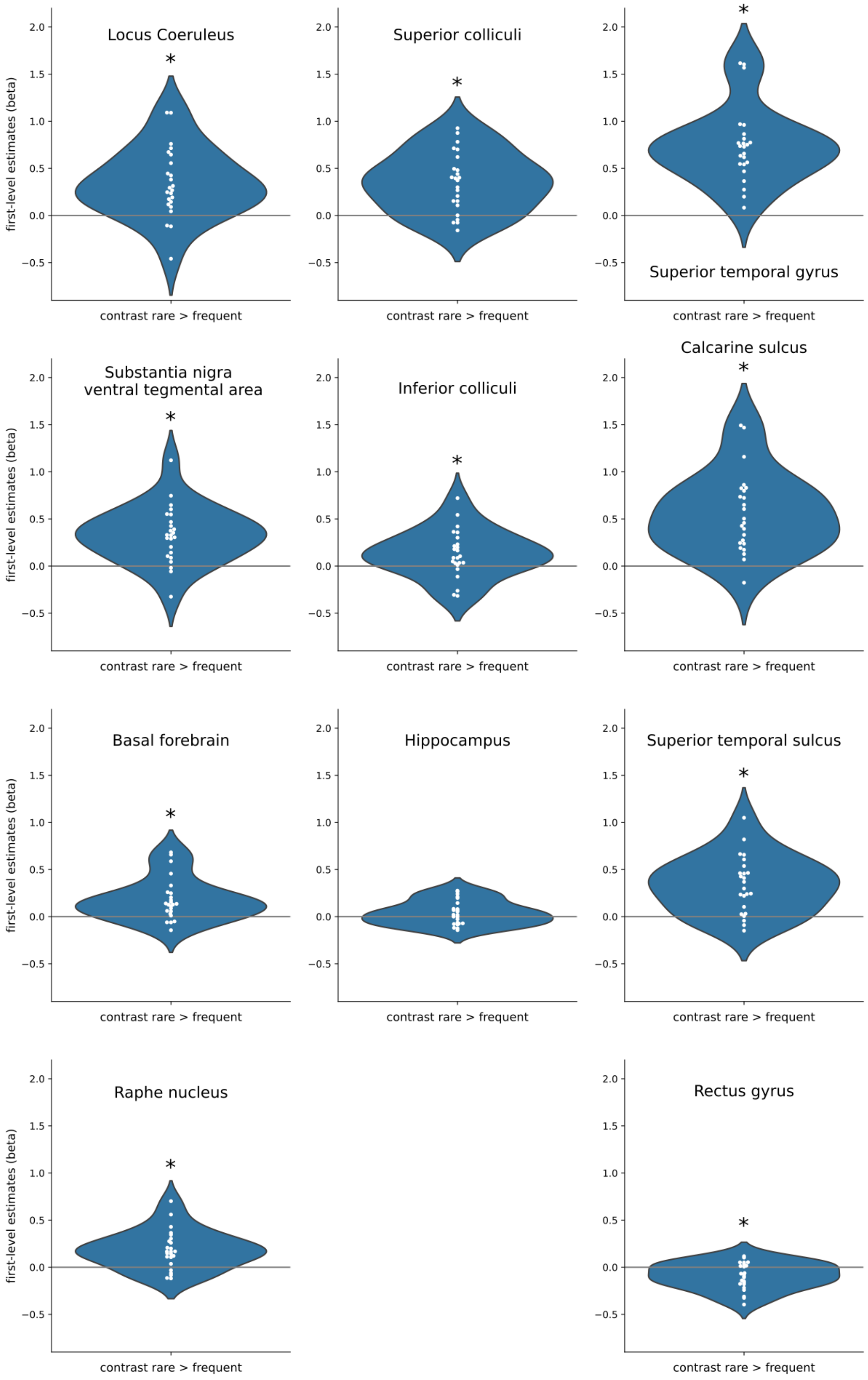

Figure S3. Single-subject estimates for the effect of rare patterns in different brain structures. The first column shows neuromodulator nuclei, the second column other subcortical ROIs, and the third column cortical ROIs. Stars indicate group-level significance ($p < 0.05$).

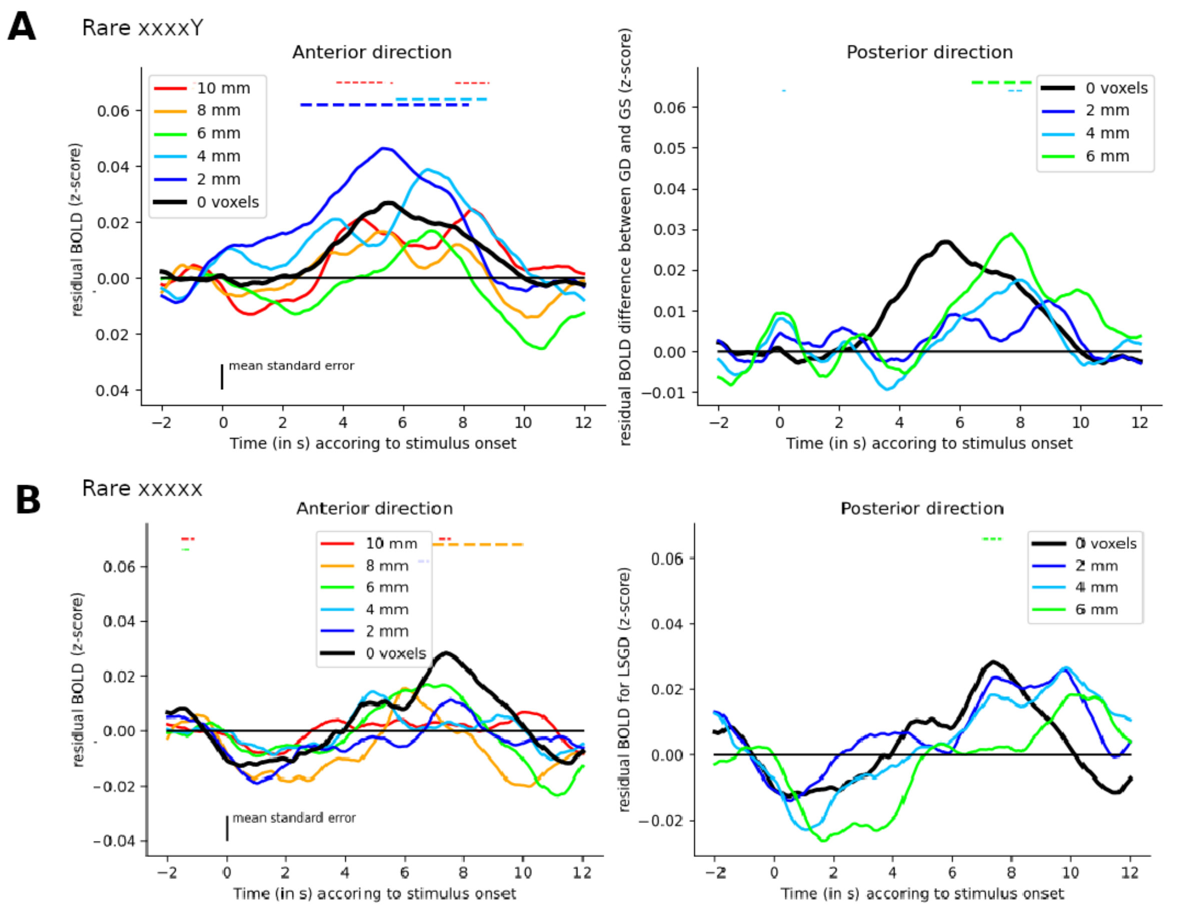

Figure S4. Time course of the z-scored fMRI activity for the two types of deviants or different shifts of the anatomically-defined LC ROI in millimeters. Two mm corresponds to 1 voxel. Colored horizontal dashed lines indicate the significant clusters ($p<0.05$; FWE corrected) identified for the difference between the corresponding color and the black line. A) For the rare xxxxY stimulus. Left panel refers to shifts toward the front of the head. Right panel refers to shifts toward the back of the head (see Figure 4B). Black line refers to the original, non-shifted LC ROI. B) For the rare xxxxx stimulus.

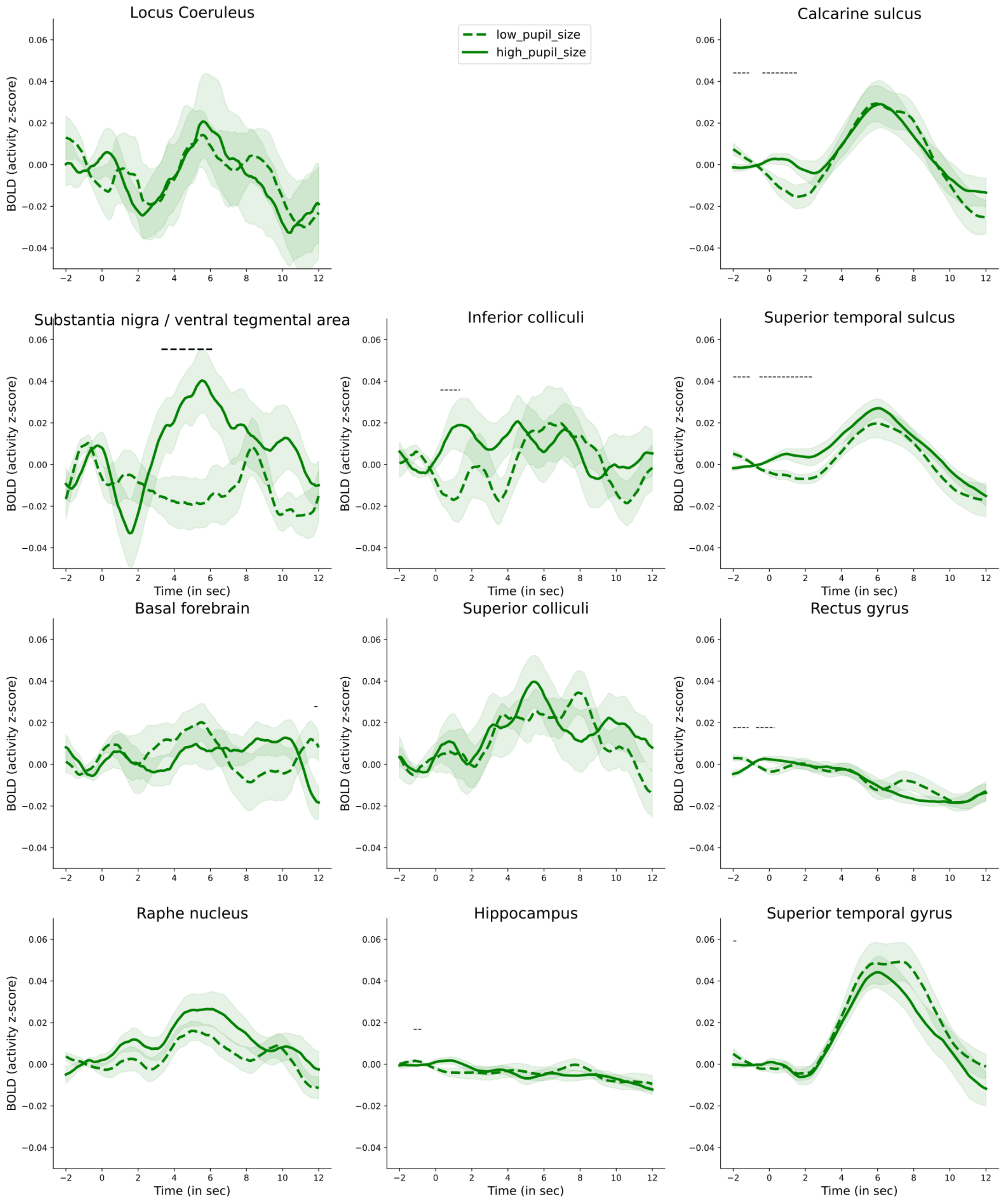

Figure S5. Time course of the effect of global deviance (z-scored fMRI activity elicited by rare), sorted by pupil response (low pupil size and large pupil size; median split), in all subcortical ROIs. Horizontal dashed black lines correspond to clusters of significant difference ($p < 0.05$). Bold dashed black lines correspond to clusters of significant difference after FWE correction ($p < 0.05$). N = 13. Mean number of included trials = 67.6 (min=29, max=88). The fMRI signal followed our prediction only in the SN/VTA ($t_{max} = 4.05$, $p_{max} = 0.002$, $d_{max} = 1.12$, cluster $p_{FWE} = 0.022$). No significant difference was found in the LC.

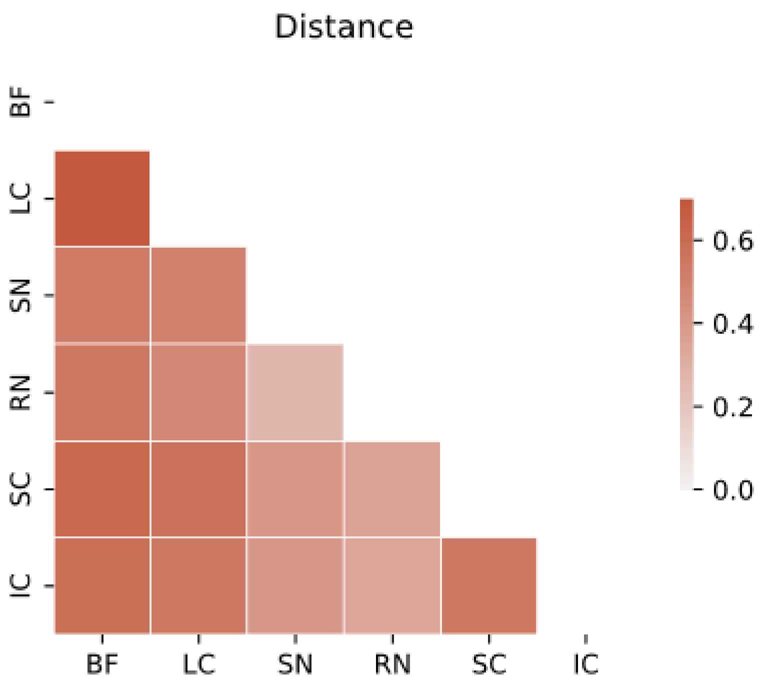

Figure S6. Distance matrix (used to make the dendrogram presented Figure 6B in the manuscript) characterizing the patterns of intrinsic correlations between each subcortical region and all cortical regions (covered in the field of view), computed for all pairs of subcortical regions. A larger distance between two regions indicates a greater difference in their patterns of intrinsic correlations with cortical regions.

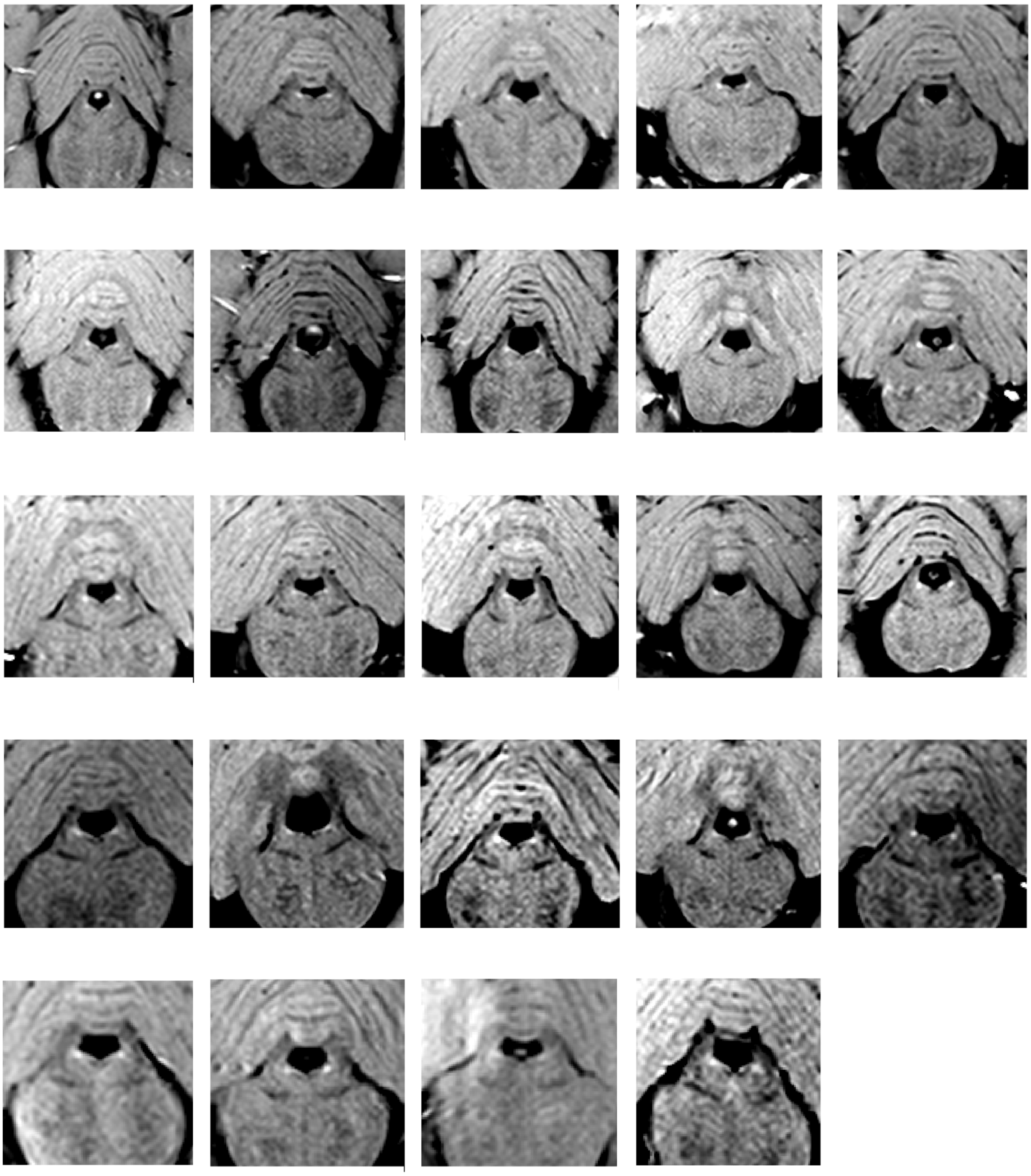

Figure S7. Anatomical images showing the LC in hypersignal for each participant.

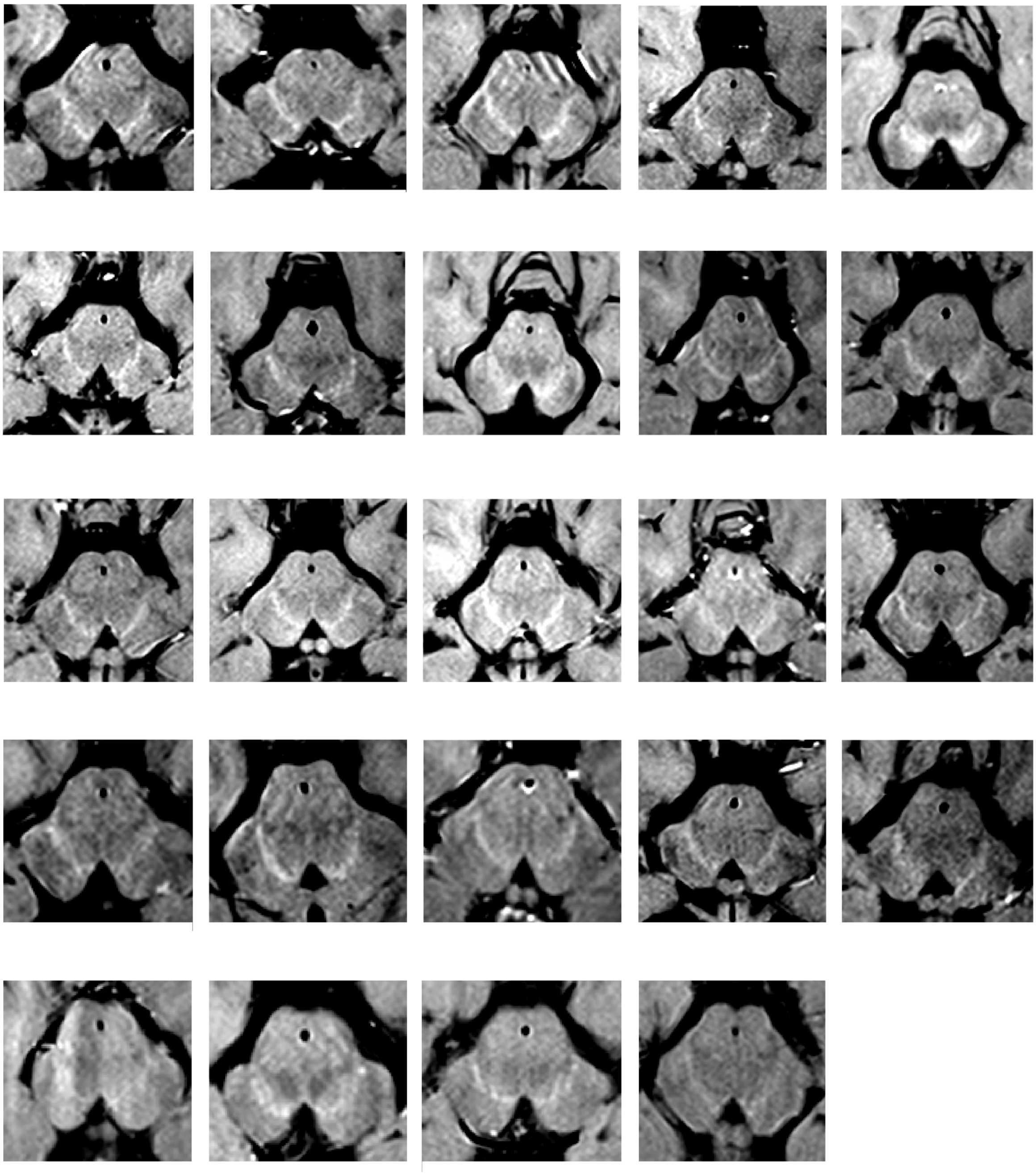

Figure S8. Anatomical images showing the SN/TVA in hypersignal for each participant.
